# Integrated Network Analysis Reveals FOXM1 and MYBL2 as Key Regulators of Cell Proliferation in Non-small Cell Lung Cancer

**DOI:** 10.3389/fonc.2019.01011

**Published:** 2019-10-15

**Authors:** Firoz Ahmed

**Affiliations:** ^1^Department of Biochemistry, University of Jeddah, Jeddah, Saudi Arabia; ^2^University of Jeddah Center for Scientific and Medical Research, University of Jeddah, Jeddah, Saudi Arabia

**Keywords:** non-small cell lung cancer, gene expression, meta-analysis, systems bioinformatics, gene network

## Abstract

**Background:** Loss of control on cell division is an important factor for the development of non-small cell lung cancer (NSCLC), however, its molecular mechanism and gene regulatory network are not clearly understood. This study utilized the systems bioinformatics approach to reveal the “driver-network” involve in tumorigenic processes in NSCLC.

**Methods:** A meta-analysis of gene expression data of NSCLC was integrated with protein-protein interaction (PPI) data to construct an *NSCLC network*. MCODE and iRegulone were used to identify the local clusters and its upstream transcription regulators involve in NSCLC. Pair-wise gene expression correlation was performed using GEPIA. The survival analysis was performed by the Kaplan-Meier plot.

**Results:** This study identified a local “driver-network” with highest MCODE score having 26 up-regulated genes involved in the process of cell proliferation in NSCLC. Interestingly, the “driver-network” is under the regulation of TFs FOXM1 and MYBL2 as well as miRNAs. Furthermore, the overexpression of member genes in “driver-network” and the TFs are associated with poor overall survival (OS) in NSCLC patients.

**Conclusion:** This study identified a local “driver-network” and its upstream regulators responsible for the cell proliferation in NSCLC, which could be promising biomarkers and therapeutic targets for NSCLC treatment.

## Introduction

Lung cancer is one of the most commonly diagnosed cancer with high mortality around the world (1). The global prevalence of lung cancer and mortality rate is rising at an alarming pace with an estimated number of newly diagnosed lung cancer was 2.1 million while the number of deaths was 1.8 million in 2018 (https://gco.iarc.fr). Based upon histology, lung cancer is divided into two classes: (i) Non-small cell lung cancer (NSCLC) which represents approximately 85–90% of all lung cancer, and (ii) Small-cell lung cancer (SCLC) which represents approximately 10–15% of the lung cancer (1). NSCLC has three major sub-classes including (a) lung squamous cell carcinoma (LUSC), (b) lung adenocarcinoma (LUAD), and (c) large cell carcinoma. However, due to lack of clinical symptom and effective diagnostic screening, the NSCLC is generally diagnosed at an advanced stage. The 5-year overall survival rate of metastatic NSCLC is 6% and has not been significantly improved in spite of having advancement in surgery, chemotherapy, and radiation (https://www.cancer.org).

Molecular profiling of NSCLC identified mutations in the tumor suppressor genes (TP53, RB1), oncogenes (EGFR, KRAS, AKT, MAPK) and translocations in oncogenes (ALK, RET, ROS1, NTRK1, NRG1), which alter the important signal-transduction pathways (2, 3). EGFR mutants have been reported more frequently in NSCLC in nonsmokers Asians patients and showed highly sensitive to therapy with EGFR tyrosine kinase inhibitors such as gefitinib and erlotinib (4, 5). Similarly, ALK rearranged gene fusion was also highly reported in NSCLC and has been proven more effective treatment with ALK-targeted inhibitors (crizotinib and alectinib) (1, 6). The genomic mutations not only alter the protein structure but also affect the expression level of genes involved in the cell division resulting in uncontrolled cell proliferation, cell survival, and NSCLC. Previous studies mainly focused on understanding the alteration in gene expression in NSCLC tumors (7), and identified overexpressed genes including CDC20 (8), CCNB1 (9), ASPM (10), and KIF4A (11), which contributes to the proliferation of tumor cells and also associated with poor prognosis. Furthermore, the role of transcription factors (TFs) including MYBL2 (12), FOXM1 (13–15), and E2F4 (16) in cell proliferation and cell survival in NSCLC has been reported. The miRNAs, a class of small non-coding RNAs which regulate gene expression at the post-transcriptional level through binding to 3′UTR of mRNA (17, 18), are also emerging as promising biomarkers for detecting NSCLC (19). However, the molecular mechanism and regulatory network of the mRNAs, TFs, miRNAs, and proteins underlying dysregulated cell division and cell proliferation in NSCLC are still largely remain unclear. Addressing these challenges are most pivotal for developing anticancer drugs and diagnostic and prognostic biomarkers for better management and personalized treatment of NSCLC.

The emergence of high-throughput genomics, transcriptomics, proteomics, and interactome data and their integrative analysis opens a new avenue for a deep understanding of etiology of cancer (20, 21). This work is focused on applying a systems bioinformatics approach to uncover interaction and regulatory mechanism of mRNAs, TFs, miRNAs, and proteins underlying cell proliferation and progression of NSCLC. Gene expression profiles have been integrated to identify the high confidence up- and down-regulated genes in the NSCLC compared to adjacent non-tumor tissues. Moreover, using the transcriptome-interactome data, *NSCLC network* was constructed and analyzed to understand the molecular mechanism underlying the development and proliferation of NSCLC. Our analysis revealed one important “driver-network” consists of 26 genes and its upstream regulators TFs FOXM1 and MYBL2 whose overexpression are associated with dysregulation of cell cycle and enhance cell proliferation in NSCLC. Furthermore, NSCLC associated miRNAs regulating the genes of “driver-network” were also identified. Combination of genes in the “driver-network” and upstream regulators could be potential biomarkers for diagnosis and prognosis; and therapeutic targets for better treatment of NSCLC.

## Materials and Methods

### Gene Expression Data Collection

In February 2019, microarray gene expression data were searched in Gene Expression Omnibus database (GEO: www.ncbi.nlm.nih.gov/geo/) using following criteria: (a) Lung cancer; (b) Human; and (c) Expression profiling by array; which gave 304 unique GEO series (GSEs). Then, a careful manually selected the GSEs data using following criteria: (d) Each GSE must have the profile of NSCLC along with adjacent non-tumor tissues as a control; (e) Each group (NSCLC/control) must have more than 20 samples; (f) All GSEs are from same microarray platform. Based upon the above criteria, three GSEs data [GSE27262 (22, 23), GSE18842 (7), and GSE19804 (24)] were selected and downloaded for further study (Table S1).

### Identification of Differentially Expressed Genes (DEGs)

This study analyzed the gene expression data having 131 samples from NSCLC and 130 samples from adjacent non-tumor tissues as control (Normal). Preprocessing of each microarray raw data including background correction, normalization and log2 transformation were performed separately with RMA of Oligo package version 1.46 in Bioconductor/R version 3.5.2 (25). Each normalized expression data was integrated into a single file and batch effects were removed with ComBat of sva package version 3.30 in R (26). After that, differential expression analysis of genes between NSCLC compared to control was calculated using the linear modeling features of the limma package version 3.38 in Bioconductor/R (27). Affymetrix probe set ids were mapped to gene symbol using DAVID 6.8 (https://david.ncifcrf.gov/) (28). The gene is considered as differentially expressed (DEGs) if log2 Fold Change |log2FC| is >2 and adjusted *P-*value is < 0.001. If multiple probe id mapped with the same gene, probe id with highest log2FC were selected. The expression data of the significant DEGs were selected and transformed into Z-score (row-wise of value), then a hierarchical clustering across rows were performed to create a heatmap using Morpheus tool (https://software.broadinstitute.org/morpheus/).

### Functional Annotation and Pathway Enrichment Analysis

In order to investigate the biological processes altered in NSCLC, we performed the functional annotation including Gene Ontology (GO) enrichment analysis for Biological Process, Molecular Function, Cellular Component, and Kyoto Encyclopedia of Genes and Genomes (KEGG: www.kegg.jp) to the list of DEGs. All these functional annotations were performed with clusterProfiler v3.10.1 in Bioconductor/R using pvalueCutoff = 0.01, pAdjustMethod = “BH,” qvalueCutoff = 0.05, minGSSize = 5 (29).

### Construction and Analysis of the NSCLC Network

To construct the *NSCLC network*, DEGs were mapped to the STRING version 11 application (30). The setting parameters of STRING were: (a) meaning of network edges (confidence); (b) active interaction sources (selected all); (c) minimum required interaction score (high confidence >0.900); (d) max number of interactors to show (1st shell, none/query protein only); (2nd shell, none). The PPI data was downloaded, and the network was visualized in Cytoscape software version (3.5.1), where each node represents a protein while the edge represents an interaction between proteins (31). We also integrated the information of differential gene expression level into the network, where the red node indicates up-regulated, while the green node indicates down-regulated expression in NSCLC compared to control. The topological structure of the *NSCLC network* was analyzed using Cytoscape plug-in “NetworkAnalyzer.”

Intending to identify highly connected local sub-networks in the *NSCLC network*, we applied the Cytoscape plug-in MCODE clustering algorithm (32). Furthermore, the biological relevance of these modules was analyzed with GO and KEGG pathway.

### Identifying the Upstream Regulator of Genes in Cluster

To identify the upstream transcription regulators of genes in MCODE clusters, Cytoscape plug-in iRegulone (V 1.3) was used at default parameters (33). Then, in house program was used to generate a matrix table where each row indicates TF while a column indicates a target gene. A Venn diagram was drawn using (http://www.interactivenn.net/).

### Expression Correlation Between Genes and TFs

To establish the relationship between genes in a cluster and its upstream regulators, pair-wise gene expression correlation was performed with GEPIA (http://gepia.cancer-pku.cn/index.html) (34). The web server integrated RNA-seq expression data from 9,736 tumors and 8,587 normal samples of the Cancer Genome Atlas (TCGA) and the Genotype-Tissue Expression (GTEx) projects. The analysis was done on default parameters: Pearson correlation coefficient; and selected LUAD and LUSC as TCGA tumor and TCGA normal dataset.

### Effect of Signature Genes on Survival in NSCLC by Kaplan-Meier Plot

The potential effect of expression of relevant genes on the overall survival (OS) was analyzed on the lung cancer patient. An online KM plotter software (http://kmplot.com/analysis/) was used to generate the Kaplan-Meier Plot on 1926 NSCLC cancer patients (LUAD and LUSC) (35). The tool run on the default parameters on hazard ratio (HR) with 95% confidence intervals and log-rank *P*-value which is considered as significant *P*-value < 0.05. The biased arrays (*n* = 2,435) were excluded for quality control.

### Extension of Cluster 1 With miRNA

In order to understand the regulatory role of miRNAs in NSCLC, the differentially expressed miRNAs (DEMs) in NSCLC compared to normal was downloaded from miRCancer database (http://mircancer.ecu.edu/) (36). After removing redundancy and cleaning of the data, 56 miRNAs were appeared as up-regulated, while 168 miRNAs were appeared as down-regulated in NSCLC compared to control. The targets of these DEMs were identified using miRNet tool (https://www.mirnet.ca/). After that, only those miRNAs were selected for further study which are targeting any of the 31 genes (*Cluster 1* and its associated TFs). Finally, an extended sub-network of *Cluster 1* was generated by integrating *Cluster 1* with its upstream regulators of TFs and miRNAs in Cytoscape. To make sparse visualization of network, interaction within *Cluster 1* as well as between *Cluster 1* and TFs were removed.

### Mutational Signatures in NSCLC

The cBio Cancer Genomic Portal (http://cbioportal.org) is a freely available tool to explore cancer genomic data in diverse cancers. We selected the NSCLC from TCGA database and submitted the list of 31 genes from *Cluster 1* and its associated TFs in cBioPortal.

## Results

### Verification of Each Group of Samples Using Principal Component Analysis

The Principal Component Analysis (PCA) was performed on normalized data of gene expression, which revealed a clear difference between NSCLC and normal samples in each GSEs study (Figures S1A–C). The cumulative contribution of PC1, PC2, and PC3 is 38.59, 33.64, and 36.47% for GSE27262, GSE18842, and GSE19804 datasets, respectively. In order to increase the statistical power to discover the DEGs, the expression data of three GSEs was integrated and technical variability and noise were removed using batch effect correction. The PCA analysis clearly showed the distinction between cancer and normal samples indicating successful removal of the batch effects on the GSEs microarray data (Figures S1D,E).

### Identification of DEGs

To identify the genes contributing to the NSCLC, differential expression analysis was conducted on gene expression data. A total of 346 DEGs including 97 up-regulated and 249 down-regulated genes were identified with |log2FC| > 2 and adjusted *P-*value is < 0.001. Among DEGs, top 10 genes showing up-regulated expression are SPP1, COL11A1, COL10A1, MMP12, MMP1, GREM1, HS6ST2, GJB2, CTHRC1, and TOP2A; while top 10 genes showing down-regulated expression are AGER, CLDN18, SFTPC, GPM6A, ADH1B, FABP4, TMEM100, CLIC5, CA4, and FAM107A (Table 1). Detail information and the complete list of DEGs is provided in Table S2A for up-regulated and Table S2B for down-regulated genes. A hierarchical cluster heatmap of DEGs across biological samples reveals distinct patterns of gene expressions in NSCLC compared to normal (Figure S2).

**Table 1:** List of top 20 differentially expressed genes is NSCLC.

**Up-regulated**			**Down-regulated**		
**Gene**	**Log2FC**	**Adj. *p*-value**	**Gene**	**Log2FC**	**Adj. *p*-value**
SPP1	4.70	4.49E-66	AGER	−5.06	2.18E-89
COL11A1	4.27	3.81E-51	CLDN18	−5.05	4.65E-54
COL10A1	4.10	1.62E-67	SFTPC	−4.53	9.44E-41
MMP12	3.95	3.41E-47	GPM6A	−4.50	6.61E-89
MMP1	3.86	1.32E-35	ADH1B	−4.34	5.76E-51
GREM1	3.71	3.70E-43	FABP4	−4.30	8.44E-65
HS6ST2	3.56	4.04E-54	TMEM100	−4.28	8.16E-57
GJB2	3.39	3.21E-46	CLIC5	−4.15	2.31E-69
CTHRC1	3.38	6.08E-62	CA4	−4.13	1.45E-85
TOP2A	3.36	4.37E-66	FAM107A	−4.12	2.82E-77
ANLN	3.25	2.25E-59	WIF1	−4.03	1.35E-40
COL1A1	3.13	9.35E-49	FCN3	−4.02	2.07E-62
PSAT1	3.05	4.96E-59	GKN2	−3.90	1.01E-56
TMPRSS4	3.00	8.65E-52	STXBP6	−3.88	9.95E-66
SPINK1	2.93	1.21E-20	CD36	−3.88	2.41E-64
CDCA7	2.90	2.04E-56	Mt1m	−3.87	2.33E-50
CST1	2.90	6.08E-42	AQP4	−3.76	2.70E-37
CXCL14	2.87	5.54E-31	SFTPA1	−3.70	2.40E-28
CEACAM5	2.83	2.54E-23	cpb2	−3.69	1.29E-48
RRM2	2.76	8.65E-52	TNNC1	−3.69	4.28E-84

### Functional Annotation and Pathway Enrichment Analysis

**Figure 1:**
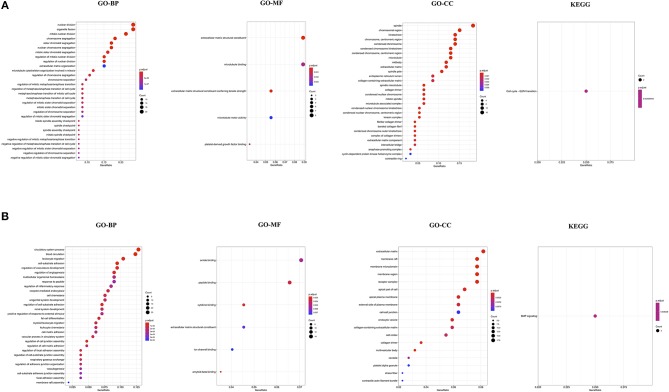
Functional annotation of up-regulated genes in NSCLC **(A)**; and down-regulated genes in NSCLC compared to control **(B)**. GO, Gene Ontology; BP, Biological Processes; MF, Molecular Function; CC, Cell Component; KEGG, Kyoto Encyclopedia of Genes and Genomes.

To understand the biological function and key pathways altered in NSCLC, function annotation and pathway enrichment analysis was performed for the list of up- and down-regulated genes. Biological process (BP) and Molecular Function (MF) of Gene Ontology analysis revealed that the up-regulated genes are primarily associated with nuclear division, organelle fission, chromosome segregation, regulation of mitotic nuclear division, metaphase/anaphase transition of cell cycle, and mitotic spindle assembly checkpoint, and microtubule binding (Figure 1A). The cellular components (CC) of up-regulated genes were significantly associated with spindle, chromosome region, kinetochore, microtubule, and midbody, fibrillar collagen trimer, and spindle microtubule (Figure 1A). The KEGG pathway analysis showed the up-regulated genes were significantly enriched in only Cell cycle-G2/M transition (Figure 1A).

Biological process (BP) and Molecular Function (MF) of Gene Ontology analysis revealed that the down-regulated genes are primarily associated with circulatory system process, leukocytes migration, cell-substrate adhesion, regulation of angiogenesis, receptor-mediated endocytosis, cell chemotaxis, regulation of cell junction assembly, amide binding, peptide binding, and cytokine binding (Figure 1B). The cellular components (CC) of down-regulated genes were significantly associated with the extracellular matrix, membrane raft, cell-cell junction, and collagen-containing extracellular matrix (Figure 1B). The KEGG pathway analysis showed the down-regulated genes were significantly enriched in only BMP signaling (Figure 1B). The complete results of GO and KEGG analyses could be found in Table S3A for up-regulated and Table S3B for down-regulated genes.

### Construction and Analysis of NSCLC Network

**Figure 2:**
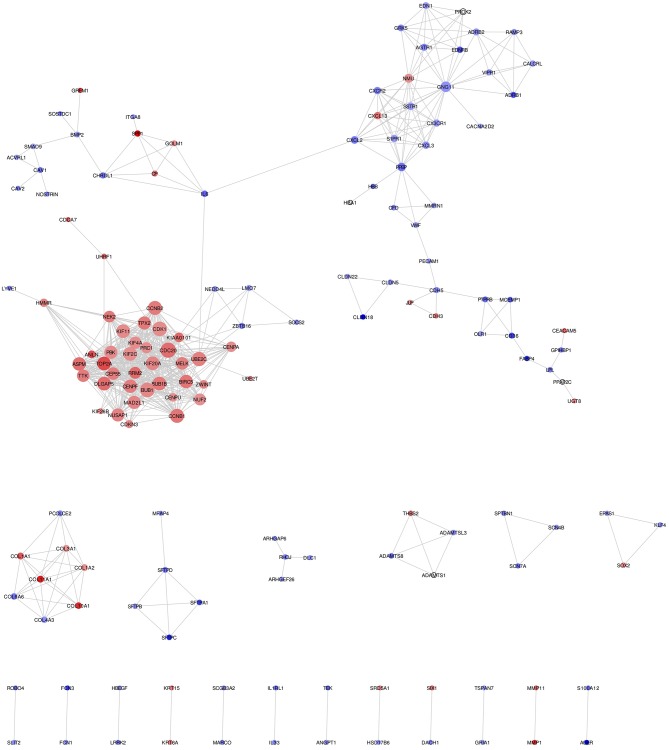
*NSCLC network* showing protein-protein integration network in NSCLC. Red node indicates up-regulated; while blue node indicates down-regulated mRNAs in NSCLC compared to normal. Size of the node is based upon degree of connectivity of the node. Edges in the network represent direct interactions between nodes.

Mapping of DEGs on STRING gave PPI network with 151 nodes and 640 edges, which were visualized in Cytoscape software, where each node represents a protein while an edge represents an interaction between proteins. The gene expression level of each protein was integrated into the PPI network, where the red node indicates up-regulated, while the blue node indicates down-regulated gene expression level in NSCLC compared to normal and termed as *NSCLC network* (Figure 2). Size of the node is based upon the degree of connectivity of the node. Edges in the network represent direct interactions between nodes. As shown in Figure 2, there are 61 and 86 nodes in the network showing up-regulation and down-regulation, respectively; while 4 nodes are not having gene expression level (identified by PPI interaction and not in the list of our DEGs). A highly interconnected sub-network of overexpressed genes could be seen in the NSCLC network. The structural topological of *NSCLC network* including Betweenness Centrality, Closeness Centrality, Clustering Coefficient, and Degree were analyzed and presented in Table S4. Furthermore, highly inter-connected 15 sub-network clusters were extracted from *NSCLC network* using Cytoscape plug-in MCODE (Figure S3; Table 2). Among them, top five clusters with the highest MCODE score were considered for further study. Topologically relevant information of a gene is given as follows:

**Table 2:** List of 15 highest score clusters identified from *NSCLC Network* by MCODE.

**Cluster**	Score (Density*#Nodes)	**# Nodes**	**# Edges**	**Node IDs**
1	25.2	26	315	CEP55, KIF20A, DLGAP5, KIF4A, RRM2, KIF11, NUF2, NUSAP1, UBE2C, CCNB1, BUB1, NEK2, TPX2, BUB1B, TOP2A, MAD2L1, PBK, CCNB2, MELK, CDC20, BIRC5, CDK1, CENPF, ASPM, KIF2C, TTK
2	10	10	45	GNG11, CXCL13, NMU, CXCL2, S1PR1, CX3CR1, CXCL3, PPBP, SSTR1, CXCR2
3	7	7	21	COL3A1, COL11A1, COL6A6, COL1A1, COL4A3, COL1A2, COL10A1
4	5.111	10	23	PROK2, VIPR1, ADRB2, ADRB1, EDNRB, EDN1, RAMP3, CALCRL, GRK5, AGTR1
5	5	5	10	GOLM1, SPP1, IL6, CHRDL1, CP
6	4	4	6	PTPRB, MCEMP1, CD36, OLR1
7	4	4	6	THBS2, ADAMTSL3, ADAMTS8, ADAMTS1
8	4	4	6	SFTPA1, SFTPC, SFTPB, SFTPD
9	3	3	3	ZWINT, CENPA, CENPU
10	3	3	3	ZBTB16, NEDD4L, LMO7
11	3	3	3	VWF, MMRN1, CFD
12	3	3	3	SCN7A, SPTBN1, SCN4B
13	3	3	3	SOX2, KLF4, EPAS1
14	3	3	3	ACVRL1, CAV1, SMAD9
15	2.8	6	7	CLDN18, CLDN5, CDH5, CLDN22, CDH3, JUP

*Hub genes:* The highly connected gene in the network is called hub gene. The node CDC20 has the highest degree of connectivity [35] in the *NSCLC network*. Other top-five hub nodes with their degree of connectivity are BUB1 [33], CDK1 [33], UBE2C [32], CCNB1 [31], and CCNB2 [31] (Table S4). It is interesting to note that all 26 nodes of *NSCLC network* act as intramodular hubs of *Cluster 1*. Therefore, we considered all genes in *Cluster 1* as hub genes as their degree of connectivity are more than 21 (Figure S3; Table S4).

*Betweenness centrality of Node:* The node RHOJ has the highest betweenness centrality of 1, which connects DLC1, ARHGEF26, and ARHGAP6 (Figure 2; Table S4). Node Interleukin-6 (IL6) has second-highest betweenness centrality of 0.611 in the *NSCLC network*, which connects 6 proteins across *three* sub-networks: *Cluster 1* (UBE2C); *Cluster 2* (CXCL2); and *Cluster 5* (SPP1, CP, GOLM1, CHRDL1) (Figure 2; Table S4).

*Top DEGs in Clusters:* It was found that the highest up-regulated gene are TOP2A (*Cluster 1*), CXCL13 (*Cluster 2*), COL11A1 (*Cluster 3*), and SPP1 (*Cluster 5*); while highest down-regulated genes are PPBP (*Cluster 2*), COL6A6 (*Cluster 3*), EDNRB (*Cluster 4*), and IL6 (*Cluster 5*).

In order to understand the functional relevance, these clusters were further analyzed using GO and pathways enrichments. The *Cluster 1* consist of 26 up-regulated gene in *NSCLC network* (Table 2). Functional annotation indicates that: (a) *Cluster 1* is significantly associated with nuclear division, spindle, microtubule binding, and protein serine/threonine kinase activity (Figure S4; Table S5); (b) *Cluster 2* is significantly associated with leukocyte migration, cell chemotaxis, G protein-coupled receptor binding, and chemokine activity; (c) *Cluster 3* is significantly associated with extracellular matrix organization and collagen trimer; (d) *Cluster 4* is significantly associated with G protein-coupled receptor signaling pathway via cyclic nucleotide second messenger; (e) *Cluster 5* is significantly associated with post-translational protein modification, cytokine activity (Figure S4).

### Upstream Regulator of Cluster

Transcription factors play crucial roles in initiation, progression, and metastasis of cancer. However, the role of TFs and their downstream target genes and their regulatory mechanisms in the development of NSCLC remains largely unknown. Therefore, each MCODE cluster was analyzed to identify the potential upstream TF regulators using the iRegulone tool.

**Figure 3:**
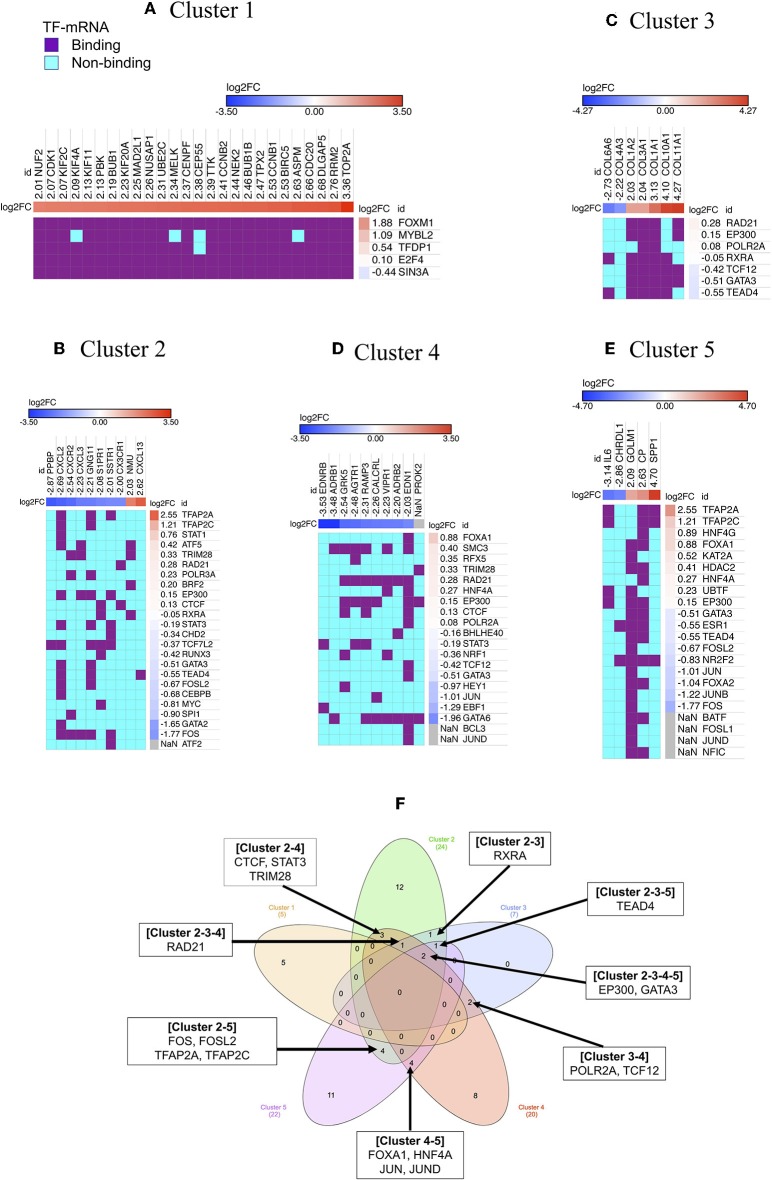
Regulators of gene cluster 1–5. Each column indicates gene in a cluster, while each row indicates TF identified by iRegulone **(A–E)**. Up-regulated DEGs in the cluster is red with positive log2FC; while down-regulated DEGs is blue with negative log2FC. TF binding with the mRNA is in purple, while non-binding in cyan. “NaN” If the log2FC is not available in our list of DEGs. **(F)** Venn diagram showing common TFs regulating different clusters.

***Cluster 1:*** Our analysis showed that almost all 26 up-regulated genes are under the control of five TFs: FOXM1, MYBL2, TFDP1, E2F4, and SIN3A (Figure 3A). However, only FOXM1 and MYBL2 are up-regulated gene showing log2FC >1, while, TFDP1 and E2F4 show slight up-regulated while SIN3A show slight down-regulated in our list of DEGs of NSCLC.

***Cluster 2:*** The cluster 2 contains 10 genes, which is under the regulation of 24 TFs, however, only four TFs showed |log2FC| >1 (TFAP2A and TFAP2C up-regulated; GATA2 and FOS down-regulated) in our list of DEGs (Figure 3B).

***Cluster 3:*** The cluster 3 contains 7 genes, which is under the regulation of 7 TFs, however, all of the TF showed |log2FC| < 1 in our list of DEGs (Figure 3C).

***Cluster 4:*** The cluster 4 contains 10 genes, which are under the regulation of 20 TFs, however, only GATA6, EBF1, and JUN showed log2FC < −1 in our list of DEGs (Figure 3D). ***Cluster 5:*** *Cluster 5* contains 5 genes, which is under the regulation of 22 TFs, however, only six TFs showed |log2FC| >1 (TFAP2A and TFAP2C up-regulated; JUN, FOXA2, JUNB, and FOS down-regulated) in our list of DEGs (Figure 3E). *Cluster 1* contains all up-regulated genes, and *Cluster 4* contains all down-regulated genes, however, the rest of the cluster contains both up-and down-regulated genes. Venn diagram showing that few TFs are commonly regulating more than one cluster (Figure 3F).

### Validation of Upstream Regulator of Cluster

A study found that the expression level of genes and their TFs are highly correlated in spite of cell diversity; while the expression level of randomly selected genes and TFs show very weak correlation (37). Therefore, TFs interacting with its potential target gene in clusters of *NSCLC network* were analyzed for their expression correlation.

**Figure 4:**
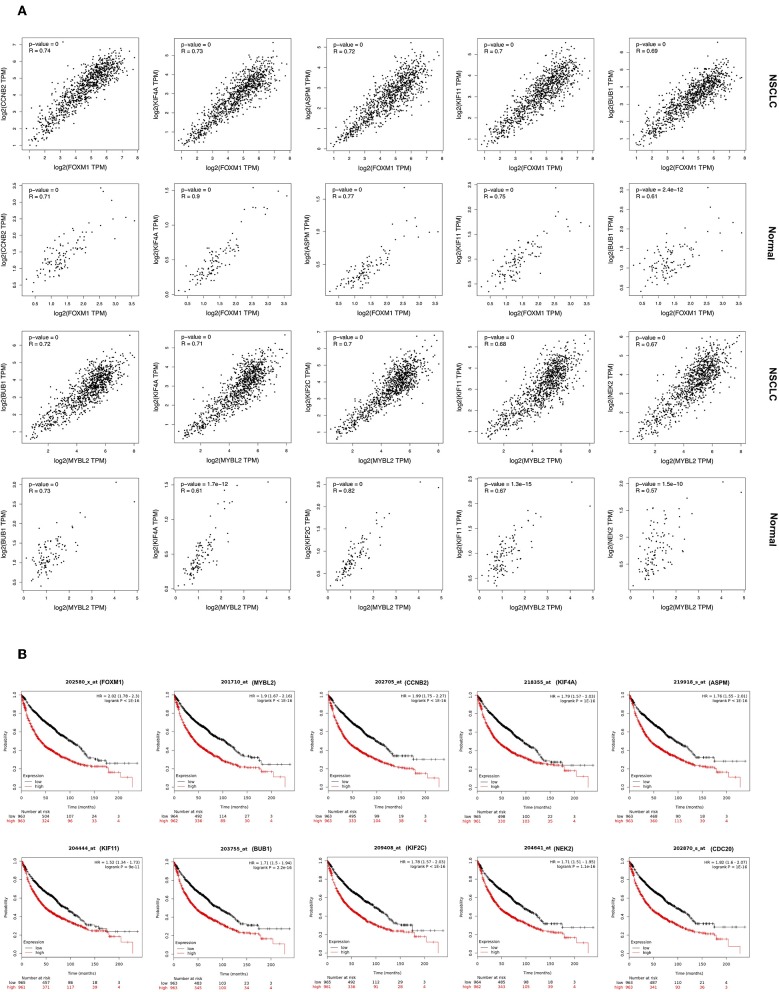
**(A)** Correlation analysis of expression of genes in *Cluster 1* and its TFs. Expression of gene is on Y-axis while TF is on X-axis. **(B)** Overall survival analysis in NSCLC patients using Kaplan-Meier plots for genes of *Cluster 1* and associated TFs.

***Cluster 1:*** Using Pearson correlation coefficients, all genes in *Cluster 1* are showing significantly highly positive correlation with upstream TFs FOXM1, and MYBL2 in NSCLC (Figure 4A; Figure S5). As revealed in the figures, their gene expression is induced in NSCLC compared to the normal sample. Top five highly correlated expressed genes with a FOXM1 are (a) CCNB2 (*R* = 0.74); (b) KIF4A (*R* = 0.73); (c) ASPM (*R* = 0.72); (d) KIF11 (*R* = 0.70); and (e) BUB1 (*R* = 0.69). Top five highly correlated expressed genes with TF MYBL2 are; (a) BUB1 (*R* = 0.72); (b) KIF4A (*R* = 0.71); (c) KIF2C (*R* = 0.70); (d) KIF11 (*R* = 0.68); and (e) NEK2 (*R* = 0.67).

***Cluster 2:*** The highest correlation of *R* = 0.53 was observed between FOS and CXCL2 (Figure S6). However, our data showed these both genes are down-regulated in NSCLC.

***Cluster 3:*** It showed the highest correlation of *R* = 0.36 between TCF12 and COL1A2 (Figure S7). However, our data showed COL1A2 is up-regulated, while TCF12 is slightly down-regulated in NSCLC.

***Cluster 4:*** The highest correlation of *R* = 0.34 was observed between GATA6 and RAMP3 (Figure S8). Our data showed these two genes are down-regulated in NSCLC.

***Cluster 5:*** Cluster 5 showed the highest correlation of *R* = 0.33 between FOXA2 and GOLM1 (Figure S9). However, our data showed GOLM1 is up-regulated, while FOXA2 is down-regulated in NSCLC.

### Gene Expression-Based Survival Analysis in NSCLC by Kaplan-Meier Plot

The topologically significant genes in the global *NSCLC network*, genes in MCODE clusters, and upstream regulator TFs (showing |log2FC|>1) were analyzed for association with OS in NSCLC using Kaplan-Meier plots. Kaplan-Meier plots of each cluster and their associated TFs are presented as follow: *Cluster 1* in Figure 4B and Figure S10; *Cluster 2* in Figure S11; *Cluster 3* in Figure S12; *Cluster 4* in Figure S13; and *Cluster 5* in Figure S14. Kaplan-Meier plots showed that high expression of all the up-regulated genes of *Cluster 1* make worse the OS [HR >1], while high expression of down-regulated gene SIN3A makes better the OS [HR <1] in NSCLC (Figure 4B; Figure S10). Kaplan-Meier plots of the gene of other clusters showed very much similar patterns that high expression of up-regulated genes make worse the OS, while high expression of down-regulated genes make better the OS in NSCLC (Figures S11–S14).

### Extension of Cluster 1 With miRNA

**Figure 5:**
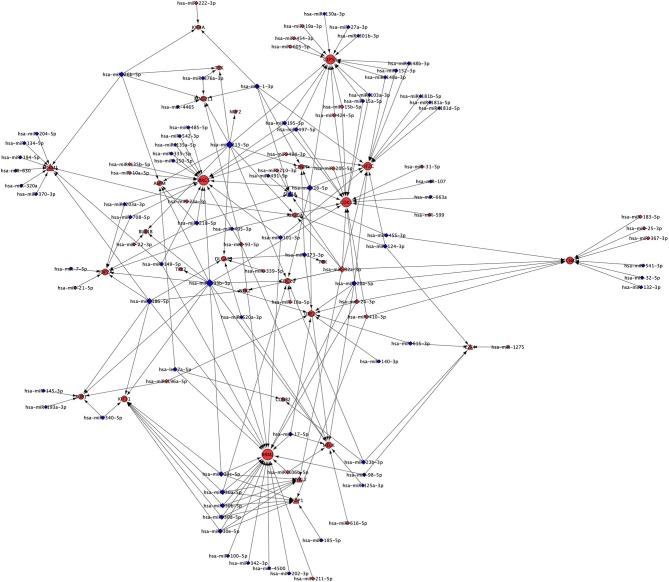
miRNA network of *Cluster 1* showing miRNAs targeting mRNAs and TFs of *Cluster 1*. Red node indicates up-regulated; while the blue node indicates down-regulated expression in NSCLC compared to normal. Size of the node is based upon degree of connectivity of the node. Nodes shape with triangle, round, and diamond represent TFs, mRNAs, and miRNAs, respectively.

Our analysis identified 30 up-regulated and 70 down-regulated miRNAs targeting 25 genes of *Cluster 1* and associated five TFs. These data were used to generate miRNA network of *Cluster 1* consisting of 130 nodes and 218 interactions (Figure 5). Our analysis found none of the miRNA is targeting NUSAP1 gene of *Cluster 1*. The top five genes targeted by highest number of down-regulated miRNAs in NSCLC are: (a) RRM2 targeted by 17 miRNAs; (b) BIRC5 targeted by 14 miRNAs; (c) CEP55 targeted by 12 miRNAs; (d) KIF2C targeted by 11 miRNAs; (e) CDK1 targeted by 9 miRNAs (Table S6). Interestingly, it was found that the expression of TFs, regulators of *Cluster 1* genes, are also under the control of miRNAs as following: (a) FOXM1 is targeted by 10 miRNA (9 down- and 1 up-regulated); (b) MYBL2 is targeted by 6 miRNAs (5 down- and 1 up-regulated); (c) TFDP1 is targeted by 7 miRNAs (6 down- and 1 up-regulated); (d) E2F4 is targeted by 5 miRNAs (4 down- and 1 up-regulated); (e) SIN3A is targeted by 6 miRNAs (4 down- and 2 up-regulated). The complete list of gene and its associated miRNAs are provided in Table S6.

The miRNA targeting highest number of genes are as following: (a) hsa-miR-193b-3p is targeting 11 genes (ASPM, BUB1, BUB1B, CDC20, CDK1, KIF11, MELK, RRM2, TOP2A, TPX2, UBE2C); (b) hsa-miR-215-5p is targeting 10 genes (ASPM, BUB1B, CDC20, CENPF, CEP55, DLGAP5, KIF20A, MAD2L1, NUF2, TTK); (c) hsa-miR-186-5p is targeting 7 genes (BUB1, DLGAP5, FOXM1, KIF11, NEK2, RRM2, TOP2A); (d) hsa-miR-16-5p is targeting 7 genes (BIRC5, CDC20, CDK1, CENPF, CEP55, KIF2C, UBE2C); and (e) hsa-miR-30a-5p is targeting 5 genes (CDC20, KIF11, MYBL2, RRM2, TFDP1). The complete list of miRNAs and their targets are provided in the Table S7. Interestingly, hsa-miR-193b-3p, hsa-miR-215-5p, hsa-miR-186-5p, hsa-miR-16-5 and hsa-miR-30a-5p are down-regulated and their targets are up-regulated in NSCLC compared to control indicating their role in the development of NSCLC.

### Mutational Signatures in NSCLC

**Figure 6:**
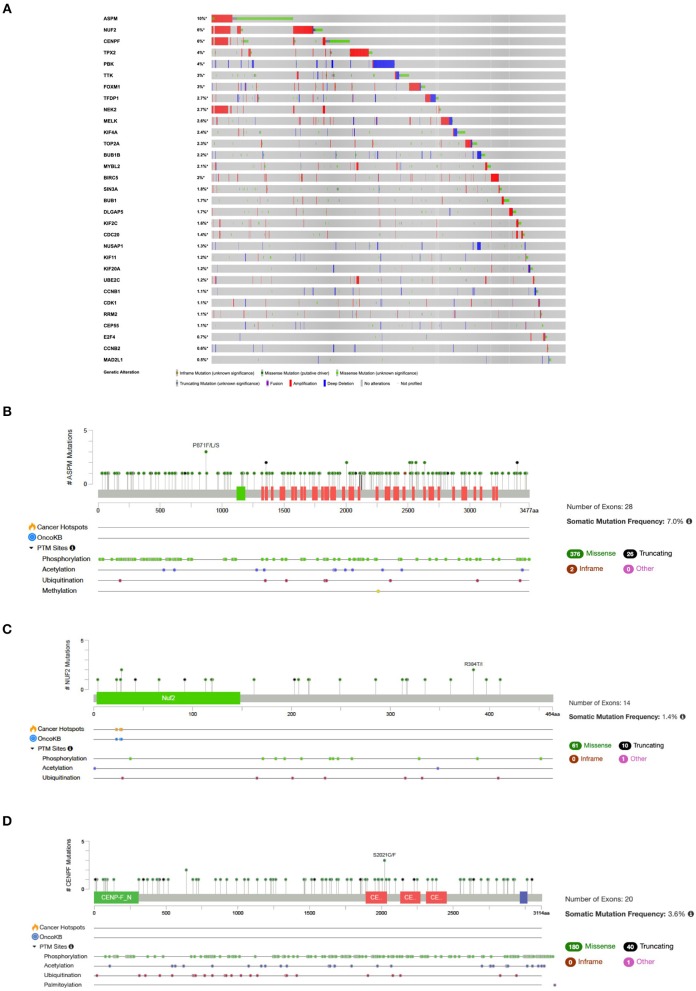
**(A)** OncoPrint of genes in *Cluster 1* and associated TFs alteration in NSCLC. Lollipop plot with distribution of mutations in NSCLC across protein domains of **(B)** ASPM; **(C)** NUF2; and **(D)** CENPF.

Analysis of mutational signatures in 31 genes (*Cluster 1* and associated TFs) in NSCLC studies showed that queried genes are altered in 1966 (37%) out of 5279 samples across TCGA datasets (Figure 6). The top three highest altered genes are ASPM (10%), NUF2 (6%) and CENPF (6%) (Figures 6A–D).

Our accumulating results indicate *Cluster 1* is working as a “**driver-network**” for the initiation of uncontrolled cell proliferation and development of NSCLC.

## Discussion

The availability of huge and diverse genome-scale molecular data provide great opportunity to integrate and analyze them to discover new mechanisms and experimentally testable models for initiation and proliferation of cancer (20, 38). Furthermore, the pan-cancer studies utilized the genomics and transcriptomics data and identified differences and commonalities in dysregulation of biological process across multiple cancer types (39, 40). NSCLC is a commonly diagnosed cancer with a high mortality rate. Previous studies identified numerous “driver-genes” as well as abnormally expressed genes and their functional enrichment associated with NSCLC (7, 22–24, 41). However, such studies lack the information of the regulatory network of abnormally expressed genes, which makes difficult to understand the molecular mechanism of development of NSCLC as well as to identify the potential therapeutic target genes. An earlier study integrated the gene expression data, DNA copy number alteration (CAN) and PPI data, and identified “driver-networks” containing potential target genes in breast cancer (20).

In the current study, a meta-analysis of large gene expression samples (131 NSCLC and 130 control) identified 346 DEGs (97 up-regulated and 249 down-regulated) in NSCLC compared to adjacent non-tumor lung tissues. After integrating the PPI data into DEGs, the *NSCLC network* was created and analyzed to understand the dysregulated sub-networks and pathways in NSCLC. Furthermore, the sub-networks were studied to identify a “driver-network” and its upstream regulators by integrating the data of TFs and miRNAs. The topologically important gene in *NSCLC network*, the “driver-network,” and its upstream regulators could be candidate genes for biomarkers and therapeutic target for NSCLC. However, selecting candidate genes for biomarker and therapeutic target requires their overexpression or underexpression have a deep connection at the molecular level for the initiation and progression of tumorigenesis. Therefore, the literature and databases mining was performed on the selected candidate genes and regulatory sub-network to understand their molecular mechanism in NSCLC.

### DEGs, Functional Annotation and Pathway Enrichment

Our study found that SPP1 is the most up-regulated gene and its high expression is associated with reducing OS (Table 1; Figure S14) and thus, support the previous finding showed enhanced expression of SPP1 in several types of tumors including NSCLC (42). Network analysis showed SPP1 is the member of *Cluster 5* and under the regulation of TFs TFAP2A and TFAP2C (Figure 3E). However, in spite of overexpression of both TFs in NSCLC, our study found no positive correlation between the expression of these TFs and SPP1 and therefore need further investigation (Figure S9). SPP1 binds to CD44 and integrin receptor in the lung cancer cell and activates the FAK/PI3K/AKT pathway which induces the secretion of vascular endothelial growth factor (VEGF) resulting in increased cell survival, cell proliferation and tumor metastasis (42). Silencing the expression of SPP1 using siRNA decreased the NSCLC tumor volume and weight in mice demonstrated it as a promising therapeutic target (43). Furthermore, our analysis showed that AGER is the highly down-regulated gene in NSCLC compared to normal tissue. AGER is a multi-ligand receptor that binds various ligands derived from a damaged cell and its up-regulation at both mRNA and protein level is associated with the majority of cancers including gastric, breast, hepatocellular, colorectal carcinoma (44, 45). However, unlike other cancers, AGER is down-regulated in NSCLC and also supported by the previous finding suggested its role as a tumor suppressor in lung cancer (46). S100A12 is a small protein express by neutrophil granulocytes and binds with AGER receptor, which induces the production of proinflammatory cytokines (47). AGER and S100A12 are interacting in *NSCLC network* and both are down-regulated suggested their role in reducing the inflammation to escape from the immune response in NSCLC (Figure 2) (47). A previous study also supports our finding that SPP1 and AGER are highly up-regulated and down-regulated, respectively, in NSCLC (48). A pan-cancer analysis of pediatric leukemias and solid tumors identified 142 and 82 driver genes (40). Comparing with pan-cancer driver genes, our study found that SIX1 was up-regulated, while TAL1 and ID4 were down-regulated in NSCLC (40). The pan-cancer study reported mutation in SIX1, TAL1, and ID4 were present in the Wilms Tumors, T-lineage acute lymphoblastic leukemias, and B-lineage acute lymphoblastic leukemias, respectively (40). Taken together the functional annotation and pathway enrichment analysis of DEGs indicated that up-regulated genes could lead to enhance tumor cell proliferation, while down-regulated genes decreased in immune cell migration in NSCLC, which are vital for uncontrolled cell division and survival in cancer, and to escape from the proper immune response (Figure 1).

### Topology of NSCLC Network

Topological properties of *NSCLC network* identified several other key proteins. Hub gene plays a key role in the proper maintaining the architecture of the biological network (49, 50). The study found that the intramodular hubs are significantly related to cell proliferation and survival time in cancer (49, 51). All of the protein of *Cluster 1* genes are highly interacting and therefore act as hub genes in the *NSCLC network* (Table S4). Top six hub genes with more than 30 degrees of connectivity are CDC20 [i.e. 35], BUB1 [33], CDK1 [33], UBE2C [32], CCNB1 [31], and CCNB2 [31] (Table S4). CDC20 regulates cell division through activating the anaphase-promoting complex/cyclosome (APC/C), which begins chromatid separation to enter into anaphase. Overexpression of CDC20 is reported in various cancer including breast cancer, cervical cancer, urinary bladder cancer, and associated with poor prognosis of ovarian tumors (52). It was reported that overexpression of CDC20 is associated with poor prognosis in NSCLC, which support our findings (Figure 4B) (8). Therefore, various studies considered CDC20 as a therapeutic target for cancer treatment (52).

The ubiquitin-conjugating enzyme E2 (UBE2C) is a member of the APC/C complex and promotes the degradation of various target proteins required for cell cycle progression. The aberrantly high expression of UBE2C was reported in various cancers. It was experimentally showed that the TF FOXM1 binds to the promoter region of UBE2C and activates its high expression in esophageal squamous cell carcinoma, which supports our finding that the FOXM1 as an upstream regulator of UBE2C of *Cluster 1* (Figure 3A) (53). CCNB1 interact with CDK1 to form a complex that phosphorylate their substrates and promotes G2/M transition in the cell cycle. *Cluster 1* contains various protein including CCNB1, which are degraded by APC/C E3 ubiquitin ligase complex. Overexpression of CCNB1 resulting in cell proliferation and was reported in various cancers including NSCLC (9). Inhibiting the expression of CCNB1 using siRNAs promotes apoptosis in colorectal cancer cells (54). BUB1 is component of spindle checkpoint for proper chromosome segregation and its up-regulation was reported in human prostate cancer (55).

Node with the highest betweenness centrality controls the flow of information between two nodes and therefore, could be crucial protein in signaling network and potential drug target to stop the flow of communication in a disease state. The node RHOJ has the highest betweenness centrality and interacts with DLC1, ARHGEF26, and ARHGAP6 and they all are down-regulated in NSCLC (Figure 2; Table S4). A study found that the encoded protein of RHOJ is activated by VEGF and may regulate angiogenesis. DLC1 encodes a Rho GTPase-activating protein that functions as a tumor suppressor and down-regulated in more than 95% of NSCLC and other cancers (56). A previous study supports the role of DLC1 as an inducer of apoptosis in NSCLC (57) and as a metastasis suppressor in breast cancer cells (58). ARHGEF26 function as guanine nucleotide exchange factors (GEFs), which catalyze the activation of RHOG by displacing GDP (from inactive enzyme) with GTP (active enzyme). ARHGAP6 involves in regulating the actin polymerization at the cell plasma membrane. Our network analysis indicates that Rho family proteins forming a complex of RHOJ- DLC1-ARHGEF26-ARHGAP6, which need detail study in NSCLC. Node IL6 has second highest betweenness centrality of 0.611 in the *NSCLC network*, which connects 6 proteins across *three* sub-networks. IL-6 is cytokines secreted during inflammation and chronic disease like cancer. It binds with interleukin-6 receptor alpha (IL-6Rα) present on the surface of T-cell, NK cell, B-cell and activates them. IL6 and its interacting partners CXCL2 and CHRDL1 are down-regulated; while other interacting partners UBE2C, SPP1, CP, GOLM1 are up-regulated in the *NSCLC network*. CXCL2 gene encodes secreted proteins and plays an important role in inflammation and immunoregulation. A study found CXCL2 role in the resistance of anti-cancer drug, anlotinib, in NSCLC (59). The overexpression of GOLM1 is reported in prostate cancer (60) and lung adenocarcinoma (61). Because of high betweenness centrality of down-regulated IL6, it may be playing a pivotal role in the down-regulation of the inflammatory response in NSCLC.

### Driver-Network and Upstream Regulators

Further analysis of *NSCLC network* identified the biologically informative 15 local cluster networks. Among them, the highest scoring *Cluster 1* identified as local “driver-network” having 26 overexpressed gene and their upstream regulators FOXM1 (log2FC = 1.88), MYBL2 (log2FC = 1.09), TFDP1 (log2FC = 0.54), E2F4 (log2FC = 0.10), and SIN3A (log2FC = −0.44) (Figure 3A). The “driver-network” is collectively associated with cell proliferation (*Cluster 1* in Figure S4). Interestingly, we observed a strong positive correlation between gene expression of each member of “driver-network” and its upstream regulators FOXM1 and MYBL2 in NSCLC (Figure 4A; Figure S5). Furthermore, NSCLC patients with their overexpression had significantly worse OS (Figure 4B; Figure S10).

The previous study found that MuvB core proteins interact with E2F4-DP1 and p130 or p107 to form a DREAM complex in G0/G1 phase of the cell cycle, which put the cell in quiescence state by globally repressing more than 800 cell cycle genes (62). When cell exit from quiescence state, MuvB core proteins dissociated from p130 and interacts with MYBL2 to form MMB (MYBL2-MuvB) complex. Subsequently, MMB recruits FOXM1 protein to form MMB-FOXM1 complex, which binds to the promoters of several cell cycle genes and activate their expression in G2/M phase responsible for mitosis (63). A study found that high expression MYBL2 gene disrupts the DREAM complex and increase the MMB complex formation and subsequently triggers the expression of the several target genes driving the cell proliferation in cancer (64). In this way, MMB complex function as opposite of the DREAM complex. A previous study identified the highly confident candidate target genes and regulatory network of DREAM and MMB-FOXM1 complexes involved in the cell cycle (63). Comparing to the study, it was found that all 26 genes of *Cluster 1* are the target of DREAM complex (63). The same study support that 24 genes of *Cluster 1* (except MELK and PBK) are the target of MMB-FOXM1 complex (63). Furthermore, the TFs FOXM1, MYBL2, SIN3A, and TFDP1 are the target gene of DREAM, but not the MMB-FOXM1 complex. Combining all these findings, our study indicates that most of the genes of *Cluster 1* are the common target for both DREAM and MMB-FOXM1 complexes. However, overexpression of MYBL2 and FOXM1 could disrupt the DREAM complex and enhance the formation of MMB-FOXM1 complex resulting high expression of cell cycle genes in *Cluster 1* which consequences uncontrolled cell proliferation and ultimately NSCLC.

FOXM1 is a member of the Forkhead box family of transcriptional factor that expresses in actively dividing cells. Several studies reported the overexpression of FOXM1 stimulates the proliferation of tumor cells during the progression of NSCLC and other types of cancers and also associated with poor overall survival (13–15, 65). A study found FOXM1 overexpressed NSCLC associated with resistance of cisplatin-based chemotherapy, and its inhibition using thiostrepton or siRNA reversed the drug resistance resulted in inhibition of cell proliferation and induce cell death (14). Silencing of FOXM1 expression by siRNA in A549 lung adenocarcinoma cells resulted in significant reduction in cell cycle-promoting cyclin A2 and cyclin B1 genes, as well as DNA replication and mitosis (13). DREAM complex directly represses the transcription of TOP2A, which encode DNA topoisomerase to relief the torsional stress during DNA transcription and replication (62). Furthermore, the study showed that depleting FOXM1 expression decrease the TOP2A mRNA and protein level in A549 human lung adenocarcinoma cells (66). Experimental studies showed the FOXM1 protein directly bind to the promoter region of TOP2A mRNA (66). Our study showed overexpression of TOP2A (log2FC = 3.36) and its upstream regulator FOXM1 indicates that both genes are the promising target for anti-cancer therapy for NSCLC (67). A pan-cancer study found FOXM1 is overexpressed across all studied 32 TCGA cancer types including NSCLC compared to normal tissues (68). A pan-cancer analysis revealed FOXM1 regulatory network as a top predictor of poor prognosis (69). We found all the genes of *Cluster 1* except NUSAP1, PBK, and CDK1 are present in the pan-cancer network associated with mitotic cell cycle and adverse prognostic genes [see Figure 2d of (69)]. In addition, the pan-cancer network contains the TFs FOXM1 and MYBL2 which support our finding.

The MYBL2 is phosphorylated by cyclin A/cyclin-dependent kinase 2 during the S-phase of the cell cycle and activate the cell division (12). Overexpression of MYBL2 is associated with poor patient survival in various cancers patient including NSCLC (12, 70). A previous study showed experimentally that several genes including KIF20A, KIF4A, NUSAP1, CCNB1, TOP2A, CDK1, CENPF, and KIF2C of *Cluster 1* are transactivated by MYBL2 (12). Abnormal expression of E2F4 and its mutations are reported in several cancers including NSCLC (16, 71, 72).

The genes of *Cluster 1* are mainly involving in the proliferation of cell division. Our study supports the previous finding that overexpression of *Cluster 1* genes in several cancer including NSCLC and associated with poor overall survival such as: KIF2C (73), KIF4A (11, 74), and KIF11 (75) which are kinesin family members of motor proteins regulating the cell mitosis through faithful chromosome condensation and segregation (76). Furthermore, it was reported that silencing of their expression using siRNAs inhibit the cancer cell growth (11, 73). Several inhibitors of KIF11 such as Monastrol, Ispinesib, and Dimethylenastron have been developed and are in clinically used to inhibit cell proliferation and induce apoptosis to treat numerous cancers (77). The high expression of ASPM responsible for mitotic spindle formation (10, 78). NEK2 (79, 80) which triggers centrosome separation are reported in NSCLC. Interestingly, another study also supports that E2F4 and FOXM1 bind to the promoter of NEK2 gene (80). NUF2 component of NDC80 Kinetochore complex regulating the chromosome segregation was overexpressed in NSCLC (81). Suppressing the expression of NUF2 inhibits tumor growth and also stimulates cell apoptosis (81). CENPF (82, 83) which is associated with the centromere-kinetochore complex and requires for chromosome segregation during mitosis.

Therefore, the “driver-network” and the predicted TFs MYBL2 and FOXM1 give more insight about the initiation and progression of NSCLC and also could be therapeutic target genes. However, a further biochemical study is required to understand the effect on cell proliferation in NSCLC by using functional siRNAs targeting a combination of TFs FOXM1, and MYBL2, and their downstream genes of “driver-network” (84).

Our study found that the “driver-network” is not only under the regulation of TFs, but also under the regulation of miRNAs. This study showed that 9 miRNAs (hsa-miR-134-5p, hsa-miR-149-5p, hsa-miR-186-5p, hsa-miR-194-5p, hsa-miR-204-5p, hsa-miR-26b-5p, hsa-miR-320a, hsa-miR-370-3p, hsa-miR-630) targeting FOXM1 are down-regulated in NSCLC. In addition, these miRNAs are also targeting 11 other genes (ASPM, BIRC5, BUB1, DLGAP5, KIF11, KIF4A, MAD2L1, NEK2, RRM2, TOP2A, and TTK), which means these are common targets for miRNAs and FOXM1 (Table S7). Previous studies found the down-regulation of has-miR-134-5p and hsa-miR-149-5p were contributing epithelial-to-mesenchymal transition (EMT), a key process of cancer metastasis, in NSCLC (85, 86). These studies also demonstrated that has-miR-134-5p and hsa-miR-149-5p act as tumor suppressors by directly binds to the 3′UTR of FOXM1 and inhibiting its expression and the EMT in NSCLC (85, 86). Accumulating evidence indicate that down-regulation of these miRNAs eliminate their suppressive effect resulting overexpression of FOXM1 and its 11 downstream target genes. The decrease expression of other miRNAs in NSCLC and their role in cell proliferation and EMT has been demonstrated in several studies including hsa-miR-194-5p (87), hsa-miR-204-5p (88), hsa-miR-26b-5p (89), hsa-miR-320a (90), hsa-miR-370-3p (91) and hsa-miR-630 (92). MYBL2 and TFDP1 are targeted by five common miRNAs (hsa-miR-30a-5p, hsa-miR-30b-5p, hsa-miR-30c-5p, hsa-miR-30d-5p, hsa-miR-30e-5p), though they belong from same miRNA family. A previous study showed the down-regulation of hsa-miR-30a-5p which directly targeting MYBL2 mRNA in NSCLC (93). Interestingly, TCGA NSCLC dataset showed higher mutation rates in the genes of “driver-network” as well as its upstream regulators FOXM1 and MYBL1 in the NSCLC (Figure 6).

Previous bioinformatics studies were mainly focused on the analysis of gene expression data to identify the DEGs, their function enrichment, the interacting hub genes in NSCLC. Ni *et al*. identified five up-regulated hub genes (TOP2A, CCNB1, CCNA2, UBE2C, and KIF20A) in NSCLC (41). Huang *et al*. identified five up-regulated hub genes (CDC20, CENPF, KIF2C, BUB1, and ZWINT) in NSCLC (94). Another study found 16 hub genes (TEK, ANGPT1, MMP9, VWF, CDH5, EDN1, ESAM, CCNE1, CDC45, PRC1, CCNB2, AURKA, MELK, CDC20, TOP2A, and PTTG1) in NSCLC (95). However, our study has following advantages compared to previous studies: (a) Current study is based upon a large dataset of NSCLC obtained from different GEO microarray dataset; (b) Identified various common DEGs detected by previous studies; (c) The DEGs were integrated with PPI, TFs and miRNAs to understand the regulatory mechanism of NSCLC initiation and progression; (d) Finally, we have identified a “driver-network” consist of 26 up-regulated hub genes and their upstream regulators (FOXM1, MYBL2, and miRNAs) involved in the proliferation of NSCLC and could serve as diagnostic and therapeutic targets to treat NSCLC.

Our study suggested that a gene could be functionally important even at a small level of overexpression such as FOXM1 and MYBL2 if they act as upstream regulators of genes of interacting proteins involved in an important biological process (20). However, our study has following limitations: (a) All the findings are based upon computational analysis using integrated data of gene expression, PPI, TFs, and miRNAs; (b) Our study did not integrate the data of gene mutations and gene copy number variations, which could abolish the cis- and trans-regulatory elements of a gene resulting aberrant gene expression and cancer (96–98); and (c) Finally, our study lacks the experimental validation and therefore need further experimental testing.

## Conclusion

In this study, potential biomarkers and therapeutic targets has been identified for NSCLC using systems bioinformatics approach on the public gene expression data. All the genes in “driver-network” (*Cluster 1)* and its upstream regulators, FOXM1 and MYBL2, which collectively overexpressed and involve in the cell proliferation, and cell division are particularly promising for further study. Furthermore, we identified several tumor suppressor miRNAs and their interacting target genes in the “driver-network.” Targeting two or more genes of the “driver-network” may be synergistic and more effective therapy against NSCLC. In our study, correlation expression, OS, and gene mutations dataset with strong statistical support were used to validate our finding. However, the biochemical study on the potential biomarkers and therapeutic targets are necessary for further validation on clinical samples.

## Data Availability Statement

This study was conducted on publicly available data on Gene Expression Omnibus (GEO https://www.ncbi.nlm.nih.gov/geo/) with accession number: GSE27262, GSE18842, and GSE19804.

## Author Contributions

FA conceptualized the whole study, designed and performed all the Bioinformatics analysis, interpreted the results, wrote, and revised the manuscript.

### Conflict of Interest

The author declares that the research was conducted in the absence of any commercial or financial relationships that could be construed as a potential conflict of interest.
