# Supplementary material for: Integrated Network Analysis Reveals FOXM1 and MYBL2 as Key Regulators of Cell Proliferation in Non-small Cell Lung Cancer
Source: Front Oncol. 2019 Oct 15;9:1011. doi: 10.3389/fonc.2019.01011 (PMC6804573; doi:10.3389/fonc.2019.01011)
Supplement: Supplementary file 1 [file Data_Sheet_1.zip › SupplementaryMaterials/Figure_S8.pdf]

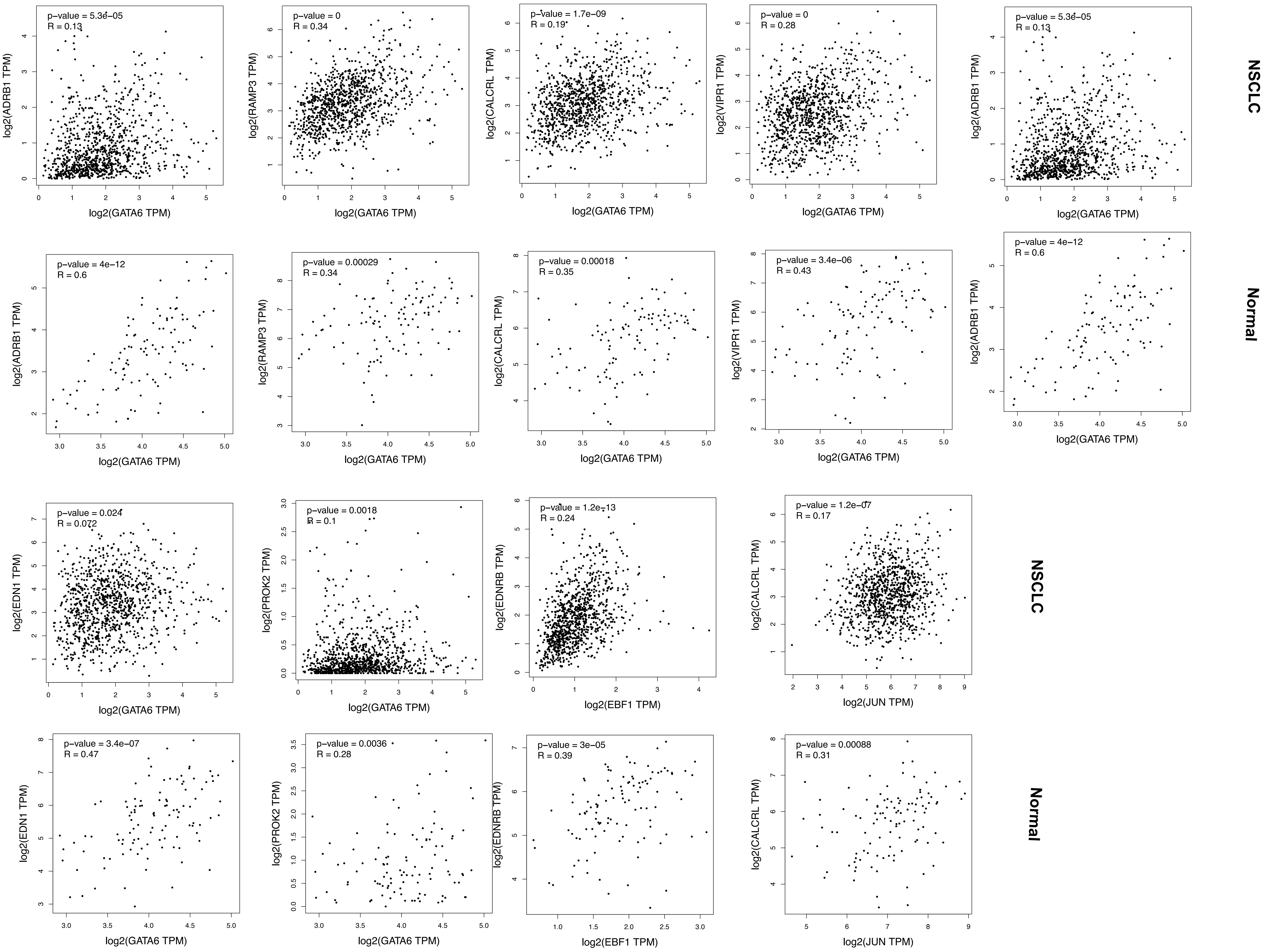

Figure S8: Correlation analysis of expression of genes in *Cluster 4* and its TFs. Expression of gene is on Y-axis while TF is on X-axis.
