# Supplementary material for: Integrated Network Analysis Reveals FOXM1 and MYBL2 as Key Regulators of Cell Proliferation in Non-small Cell Lung Cancer
Source: Front Oncol. 2019 Oct 15;9:1011. doi: 10.3389/fonc.2019.01011 (PMC6804573; doi:10.3389/fonc.2019.01011)
Supplement: Supplementary file 1 [file Data_Sheet_1.zip › SupplementaryMaterials/Figure_S11-S14.pdf]

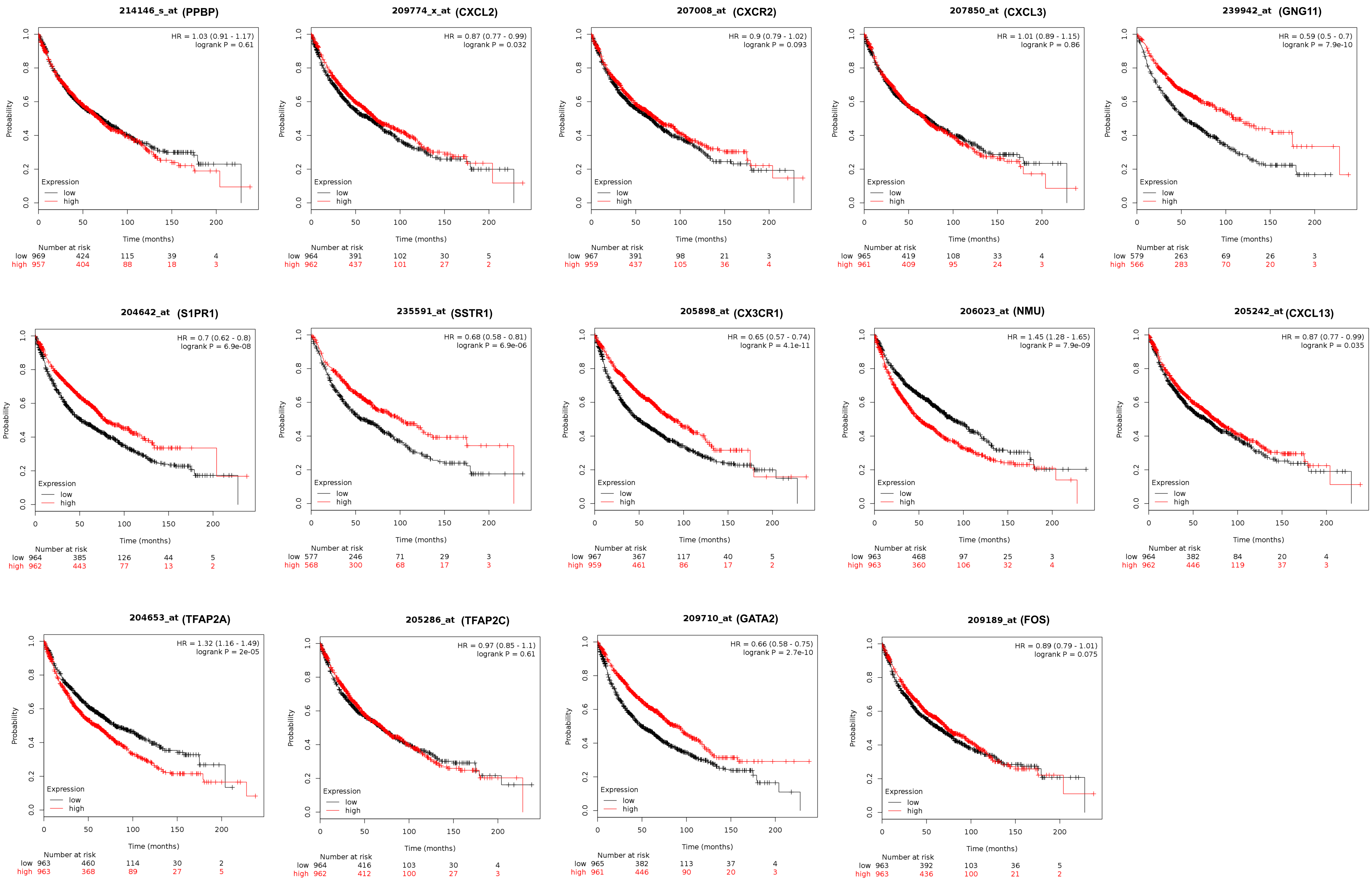

Figure S11: Overall survival analysis in NSCLC patients using Kaplan-Meier plots for genes of Cluster 2 and associated TFs.

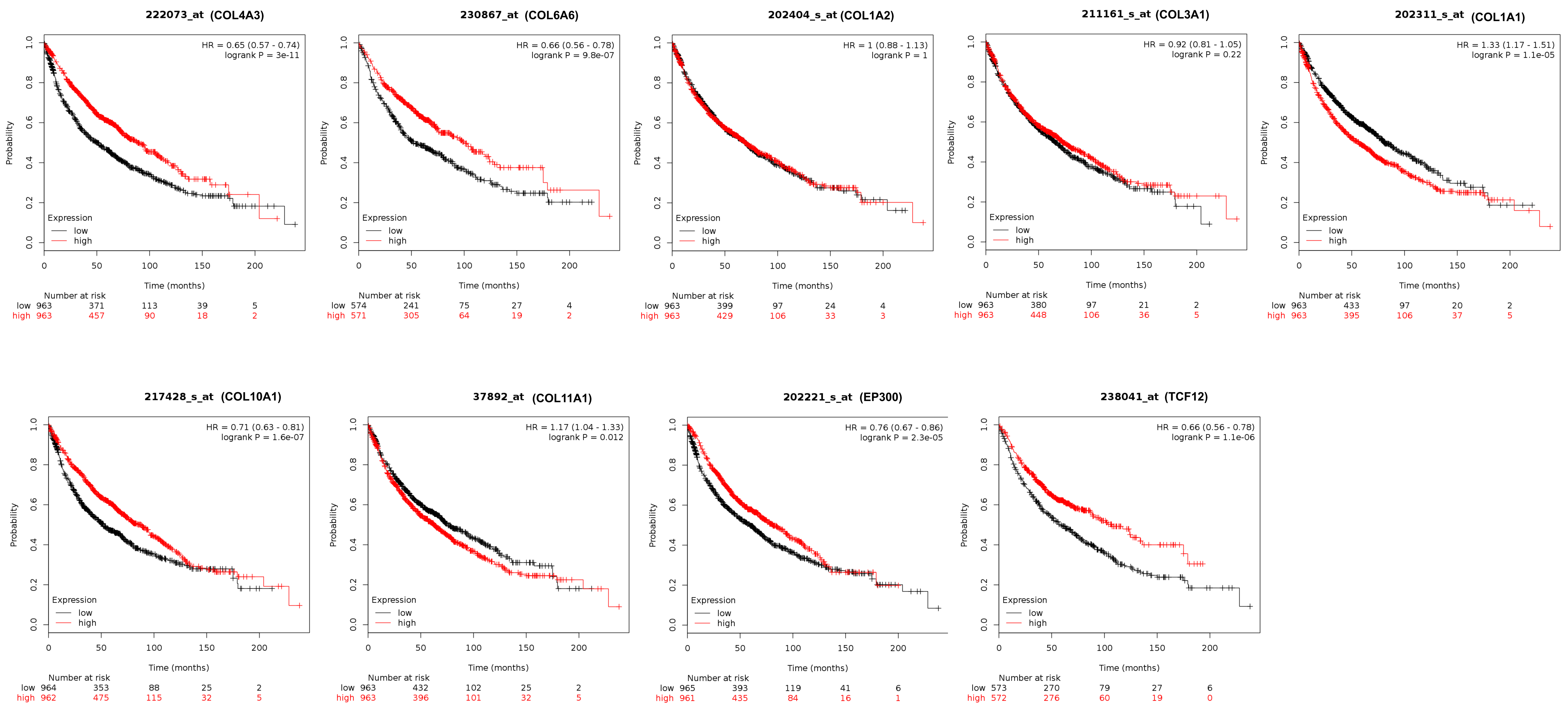

Figure S12: Overall survival analysis in NSCLC patients using Kaplan-Meier plots for genes of Cluster 3 and associated TFs.

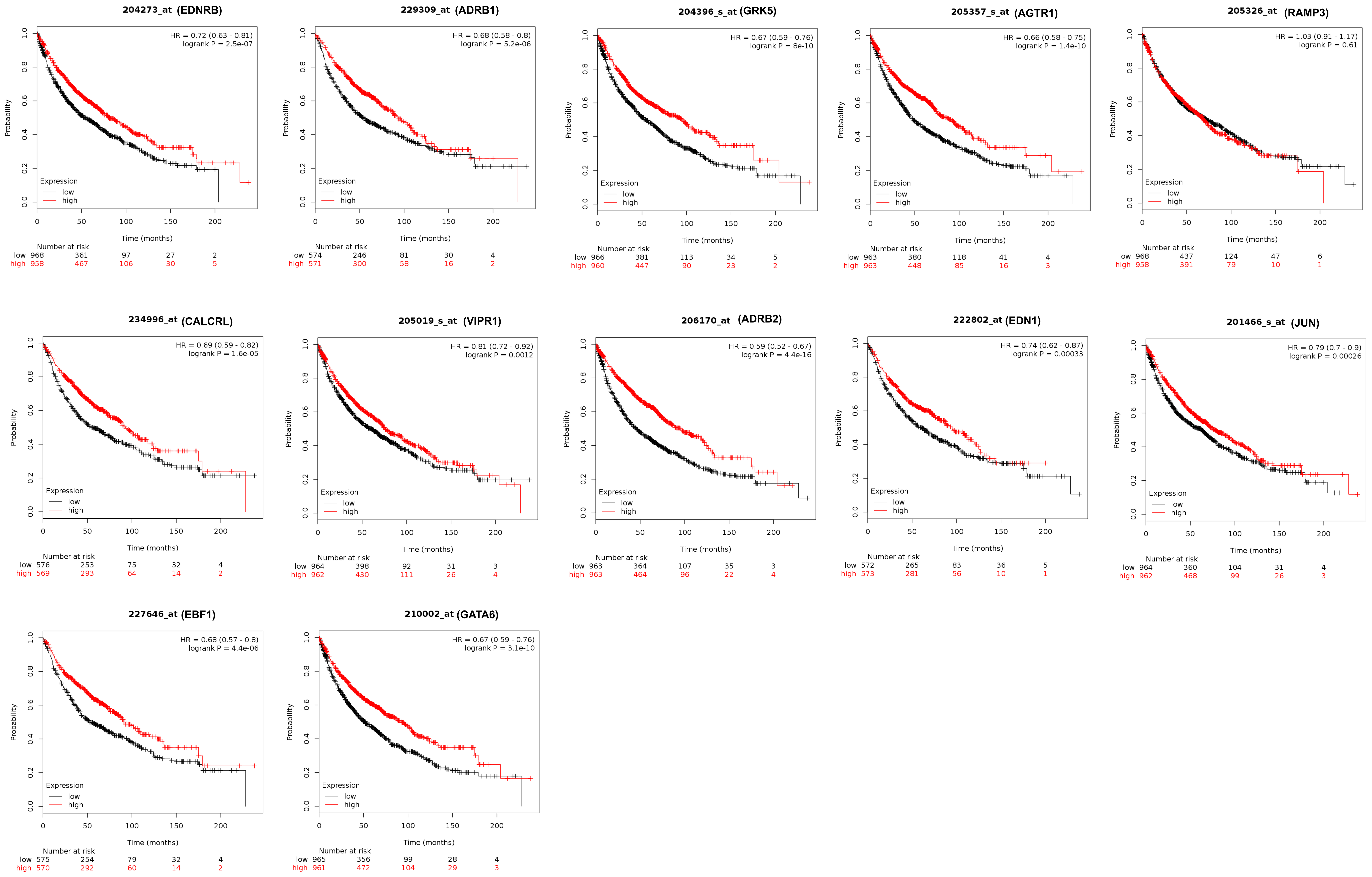

Figure S13: Overall survival analysis in NSCLC patients using Kaplan-Meier plots for genes of Cluster 4 and associated TFs.

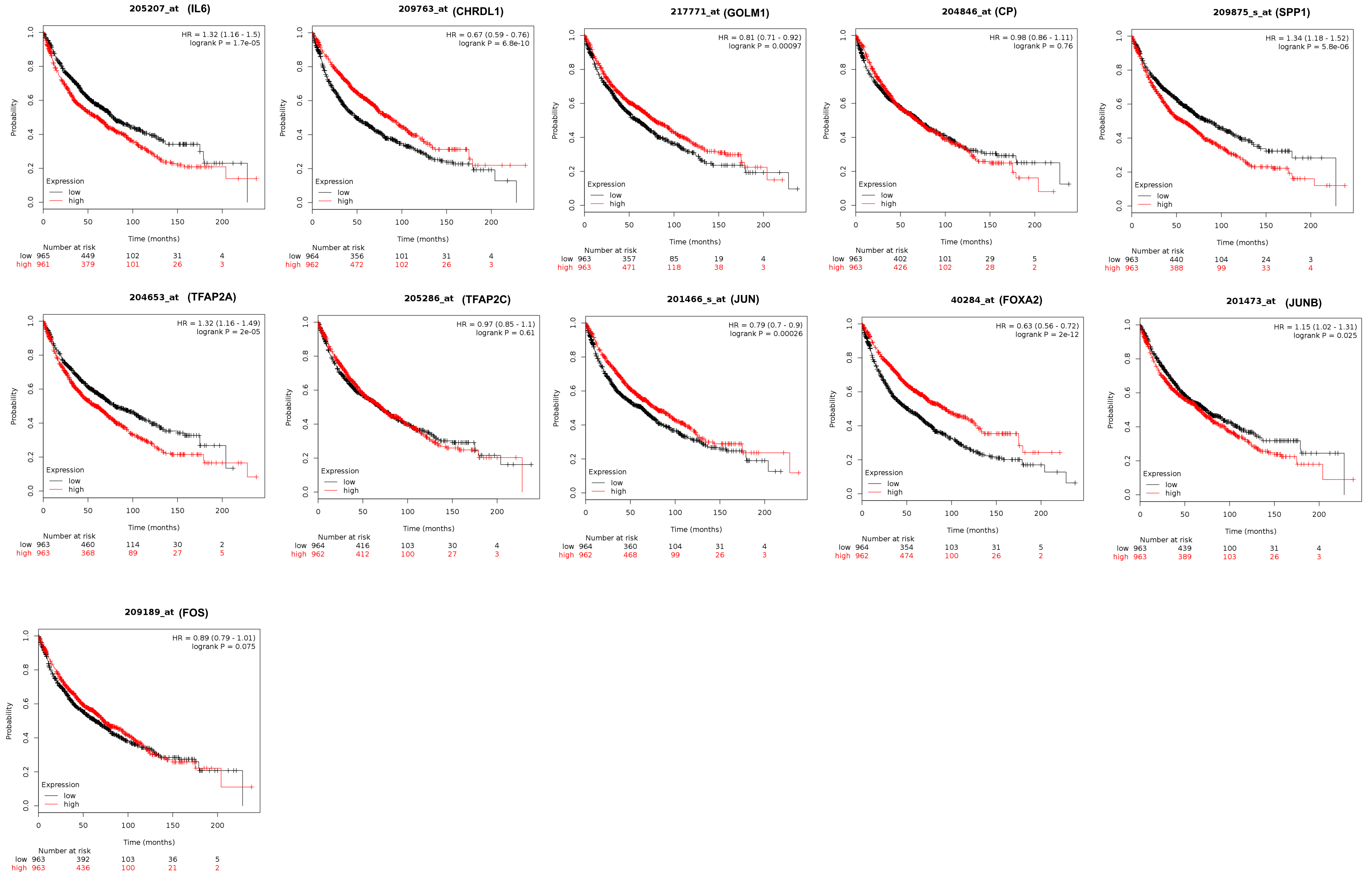

Figure S14: Overall survival analysis in NSCLC patients using Kaplan-Meier plots for genes of Cluster 5 and associated TFs.
