# Supplementary material for: Integrated Network Analysis Reveals FOXM1 and MYBL2 as Key Regulators of Cell Proliferation in Non-small Cell Lung Cancer
Source: Front Oncol. 2019 Oct 15;9:1011. doi: 10.3389/fonc.2019.01011 (PMC6804573; doi:10.3389/fonc.2019.01011)
Supplement: Supplementary file 1 [file Data_Sheet_1.zip › SupplementaryMaterials/SupplementaryInformation.docx]

**Supplementary Information**

**Integrated network analysis reveals FOXM1 and MYBL2 as key regulators of cell proliferation in non-small cell lung cancer**

*Firoz Ahmed^1,2 *^*

^1^ Department of Biochemistry, University of Jeddah, PO Box 80327, Jeddah, Saudi Arabia

^2^ University of Jeddah Center for Scientific and Medical Research, University of Jeddah, PO Box 80327, Jeddah, Saudi Arabia

^*^ To whom correspondence should be addressed.

Email (FA): [fahmed1@uj.edu.sa](mailto:fahmed1@uj.edu.sa)

| **Table S1**. List of gene expression microarray data sets used in this study. | | | | | |
| --- | --- | --- | --- | --- | --- |
| **SN** | **GEO accession**  **number** | **Sample Size** | | **Platform** | **References**  **(PMID)** |
|  |  | **NSCLC** | **Control** |  |  |
| 1 | GSE27262 | 25 | 25 | [HG-U133_Plus_2] Affymetrix Human Genome U133 Plus 2.0 Array | [25277535](https://www.ncbi.nlm.nih.gov/pubmed/25277535)  [22726390](https://www.ncbi.nlm.nih.gov/pubmed/22726390) |
| 2 | GSE18842 | 46 | 45 | [HG-U133_Plus_2] Affymetrix Human Genome U133 Plus 2.0 Array | 20878980 |
| 3 | GSE19804 | 60 | 60 | [HG-U133_Plus_2] Affymetrix Human Genome U133 Plus 2.0 Array | 20802022 |

**Figure S1:** Principal component analysis of microarray expression data of NSCLC cancer and normal. The figure showed first three PCA data (PC1, PC2 and PC3) which reveals that the gene expression patterns of NSCLC and normal are relatively distinct in each GSEs: GSE19804 (A); GSE18842 (B); GSE27262 (C). PCA before (D); and after removing batch effect (E) with ComBat of sva in R. NSCLC cancer is in red; while normal is in blue.

**Figure S2.** Heatmap of expressed gene across biological samples with hierarchical cluster. Only expression level of genes showing DEGs are included. The normalized expression value was transformed into Z-scores across each row with bright red indicating high values and bright blue indicating low values.

**Figure S3**: MCODE clusters from the NSCLC network. Top 15 highly connected gene clusters were identified from the network using MCODE. Red node represents up-regulated gene; while blue node represents down-regulated gene in NSCLC compared to normal. MCODE score is given in the bracket.

**Figure S4:** Functional annotation of MCODE five top clusters extracted from NSCLC Network. Cluster 1, Cluster 2, Cluster 3, Cluster 4, and Cluster 5. GO: Gene Ontology; BP: Biological Processes; MF: Molecular Function; CC: Cell Component; KEGG: Kyoto Encyclopedia of Genes and Genomes. Dot plot is not available for Cluster having no significant functional enrichment.

**Figure S5:** Correlation analysis of expression of genes in *Cluster 1* and its TFs. Expression of gene is on Y-axis while TF is on X-axis.

**Figure S6:** Correlation analysis of expression of genes in *Cluster 2* and its TFs. Expression of gene is on Y-axis while TF is on X-axis.

**Figure S7:** Correlation analysis of expression of genes in *Cluster 3* and its TFs. Expression of gene is on Y-axis while TF is on X-axis.

**Figure S8:** Correlation analysis of expression of genes in *Cluster 4* and its TFs. Expression of gene is on Y-axis while TF is on X-axis.

**Figure S9:** Correlation analysis of expression of genes in *Cluster 5* and its TFs. Expression of gene is on Y-axis while TF is on X-axis.

**Figure S10:** Overall survival analysis in NSCLC patients using Kaplan-Meier plots for genes of Cluster 1 and associated TFs.

**Figure S11:** Overall survival analysis in NSCLC patients using Kaplan-Meier plots for genes of Cluster 2 and associated TFs.

**Figure S12:** Overall survival analysis in NSCLC patients using Kaplan-Meier plots for genes of Cluster 3 and associated TFs.

**Figure S13:** Overall survival analysis in NSCLC patients using Kaplan-Meier plots for genes of Cluster 4 and associated TFs.

**Figure S14:** Overall survival analysis in NSCLC patients using Kaplan-Meier plots for genes of Cluster 5 and associated TFs.
