# Supplementary material for: Integrated Network Analysis Reveals FOXM1 and MYBL2 as Key Regulators of Cell Proliferation in Non-small Cell Lung Cancer
Source: Front Oncol. 2019 Oct 15;9:1011. doi: 10.3389/fonc.2019.01011 (PMC6804573; doi:10.3389/fonc.2019.01011)
Supplement: Supplementary file 1 [file Data_Sheet_1.zip › SupplementaryMaterials/Figure_S3.pdf]

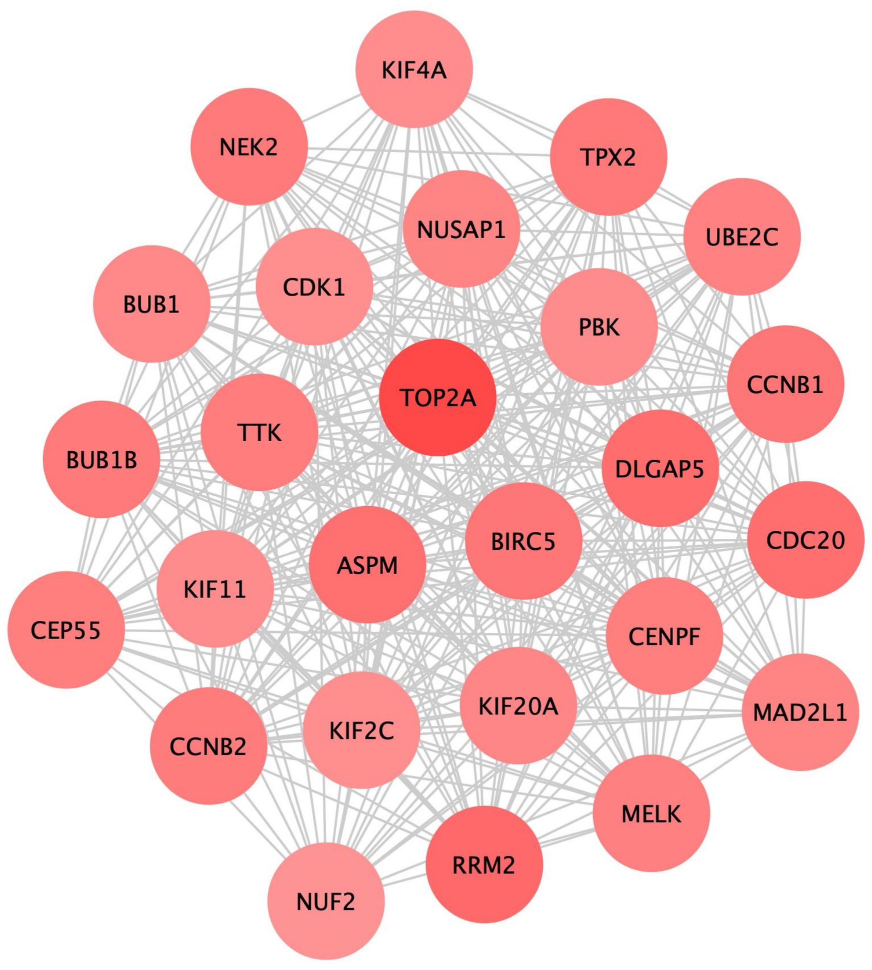

Cluster 1
(Score 25.2)

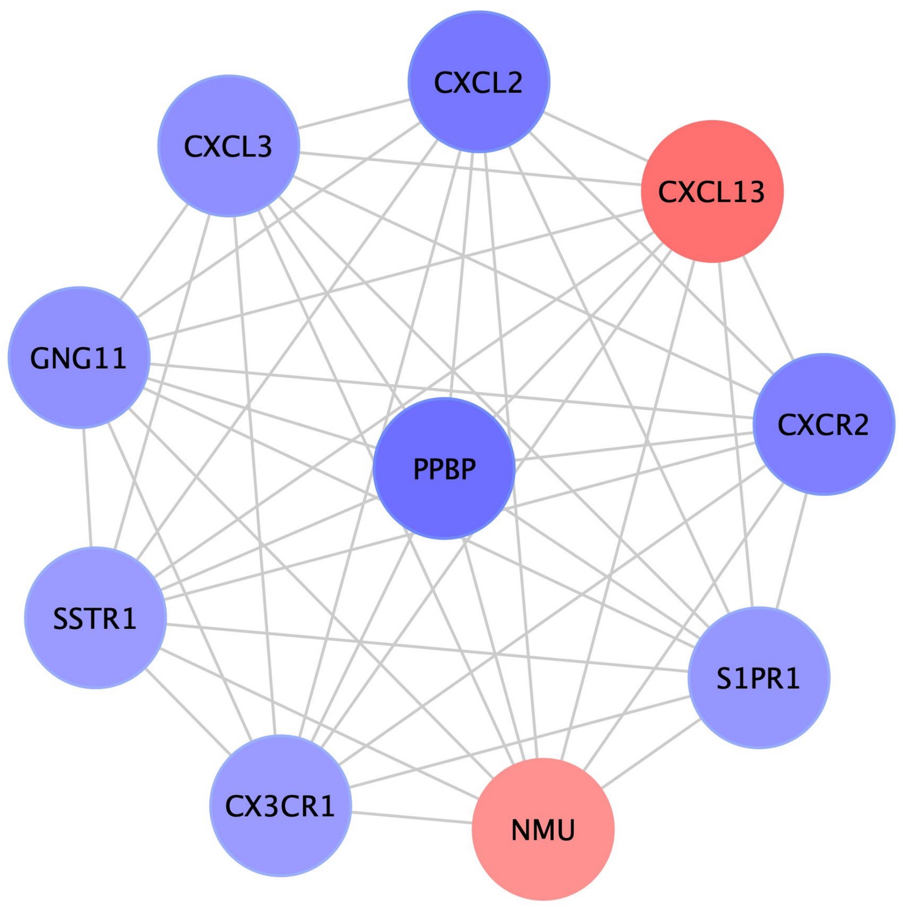

Cluster 2
(Score 10)

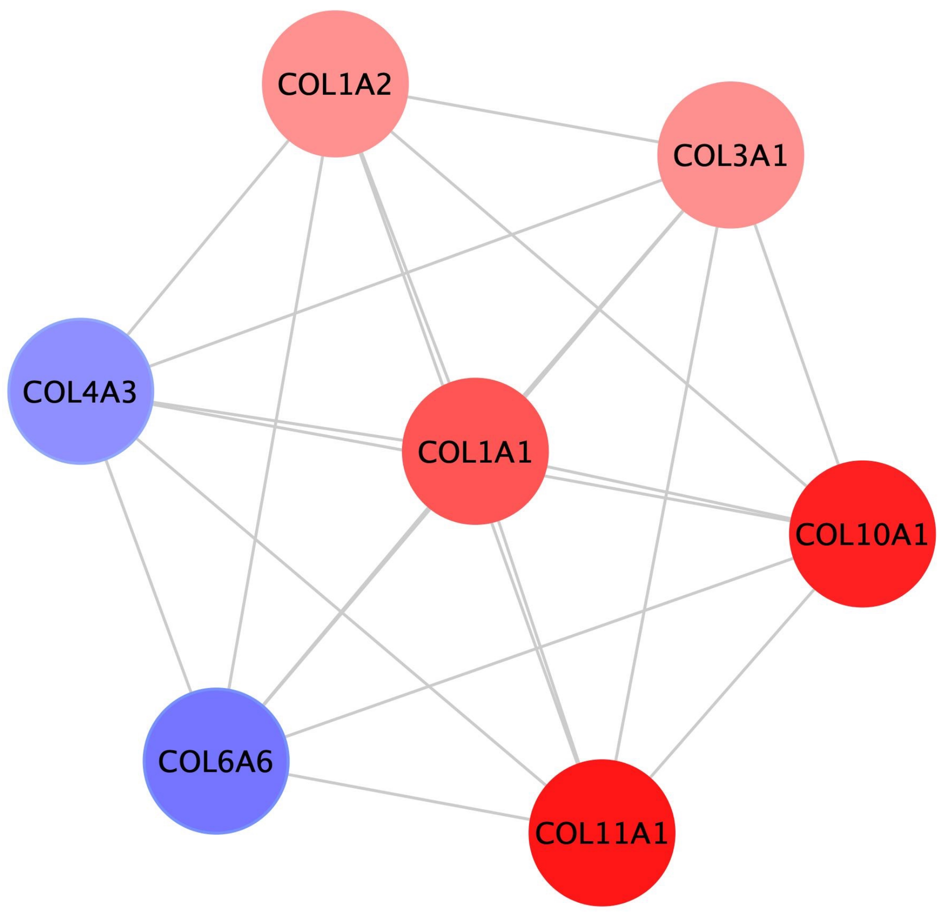

Cluster 3
(Score 7)

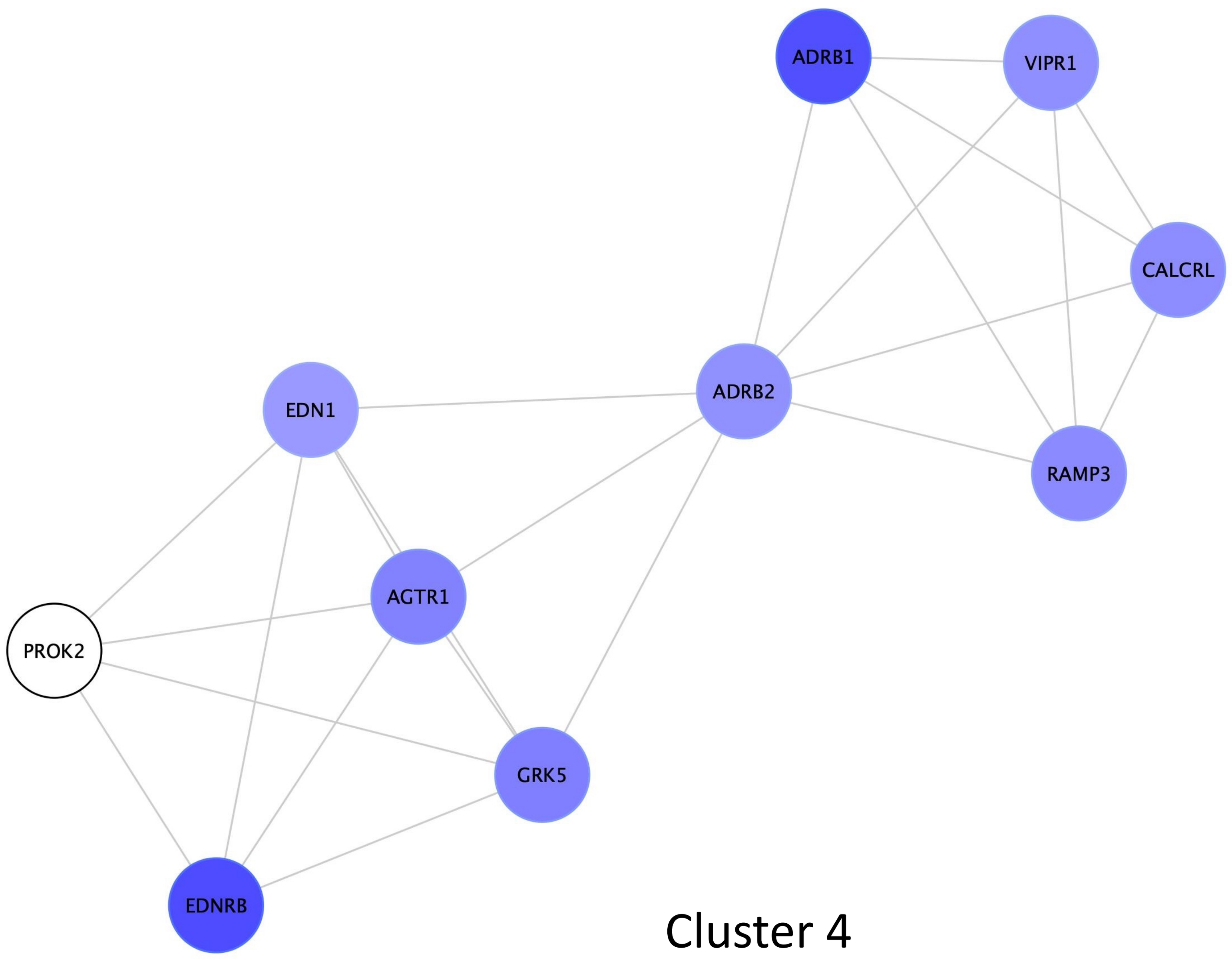

Cluster 4
(Score 5.111)

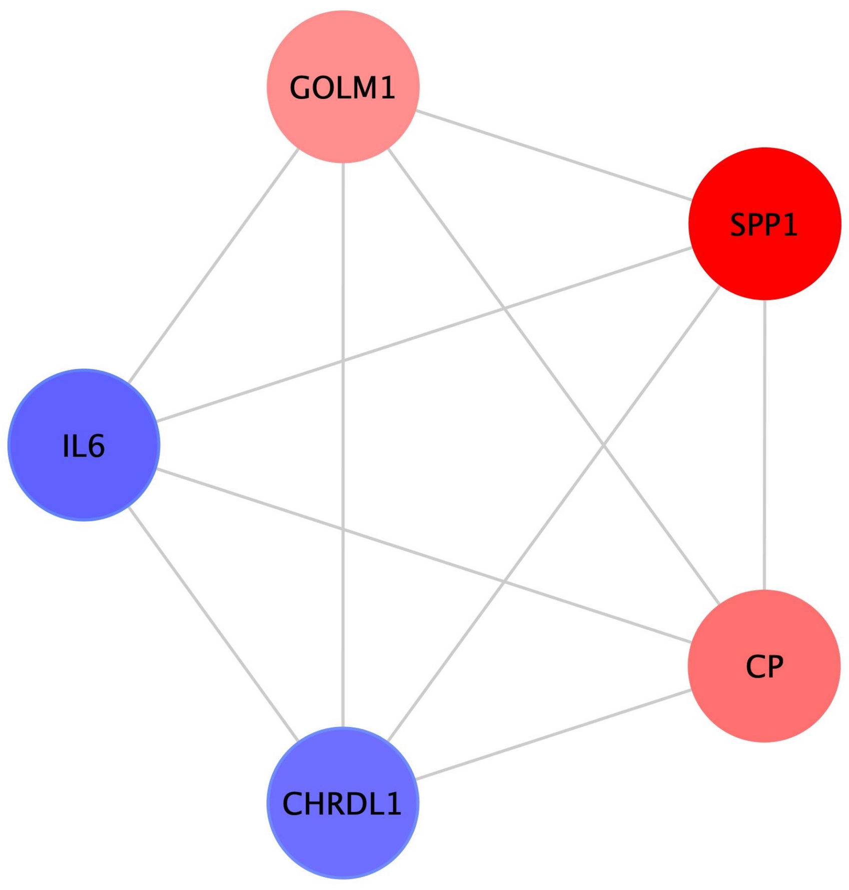

Cluster 5
(Score 5)

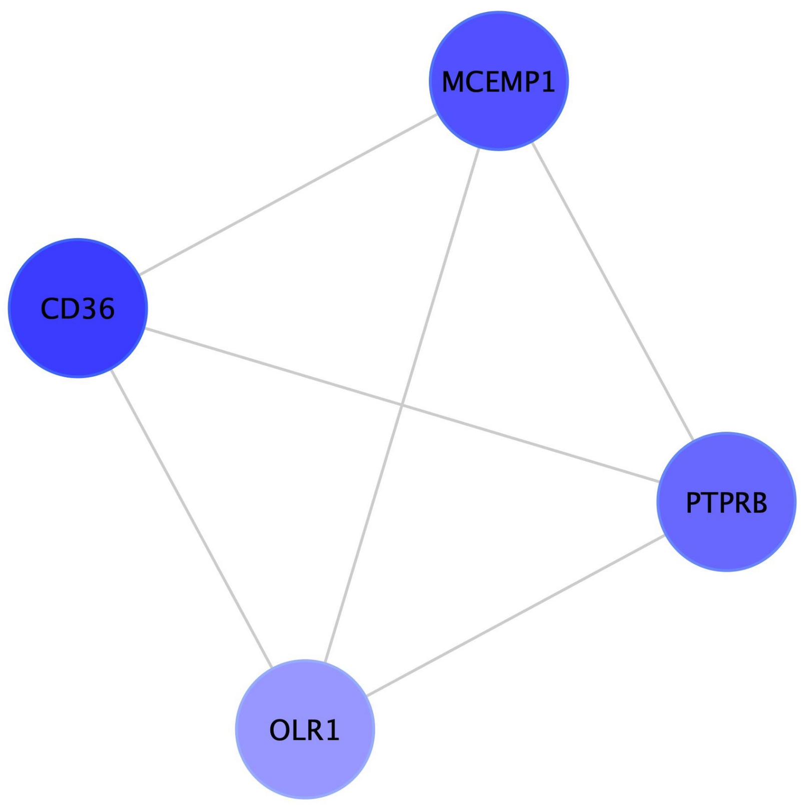

Cluster 6
(Score 4)

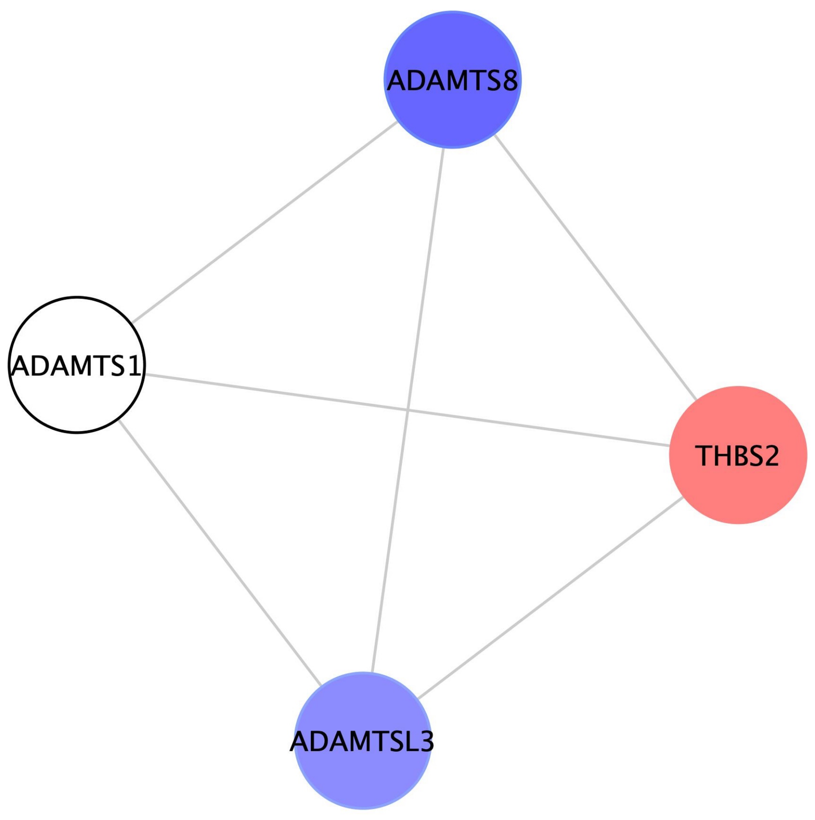

Cluster 7
(Score 4)

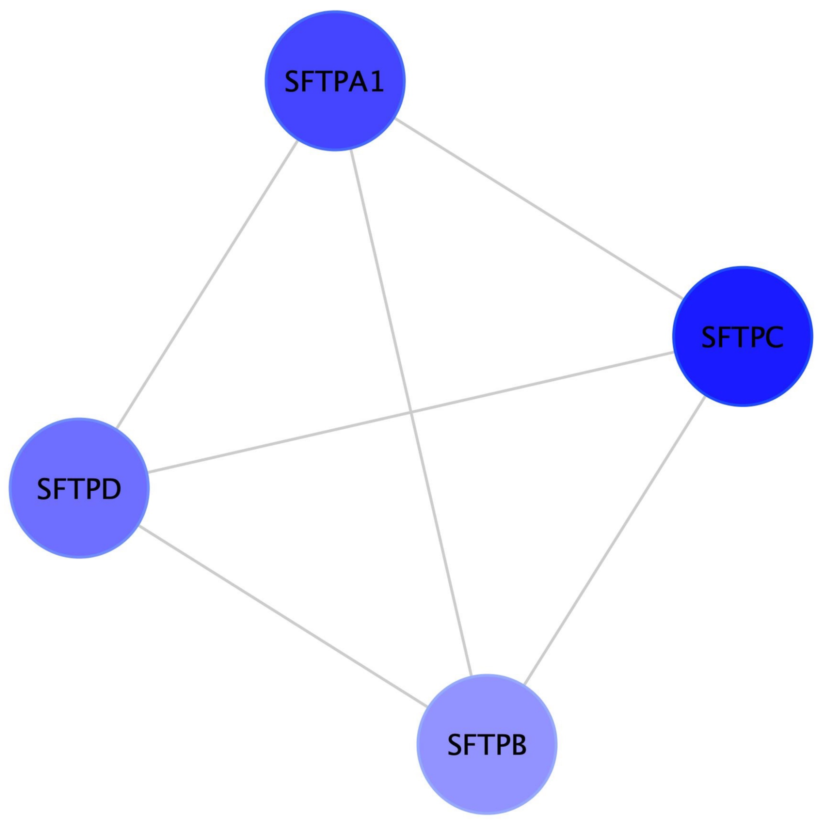

Cluster 8
(Score 4)

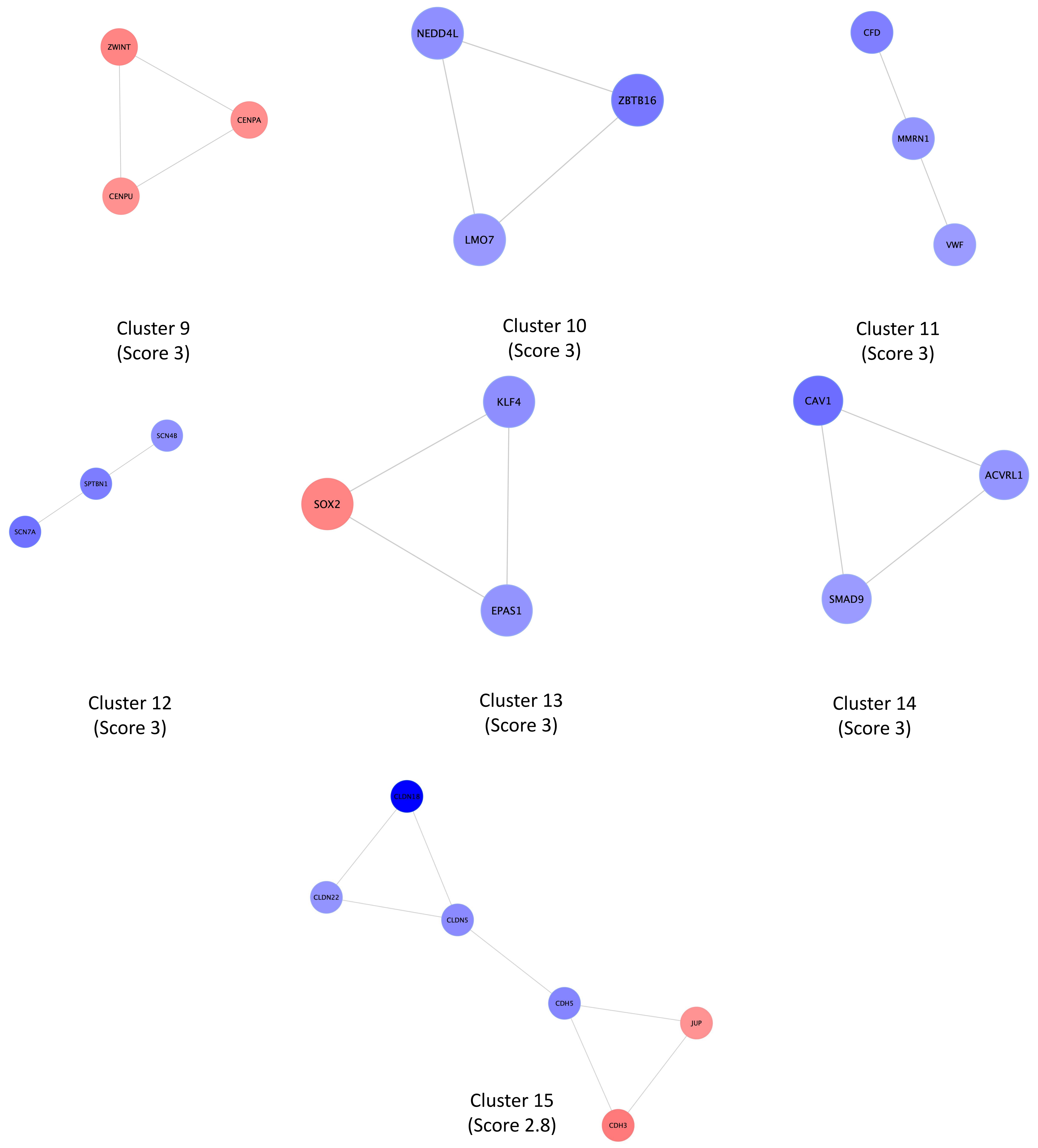

Figure S3: MCODE clusters from the NSCLC network. Top 15 highly connected gene clusters were identified from the network using MCODE. Red node represents up-regulated gene; while blue node represents down-regulated gene in NSCLC compared to normal. MCODE score is given in the bracket.
