# Supplementary material for: Integrated Network Analysis Reveals FOXM1 and MYBL2 as Key Regulators of Cell Proliferation in Non-small Cell Lung Cancer
Source: Front Oncol. 2019 Oct 15;9:1011. doi: 10.3389/fonc.2019.01011 (PMC6804573; doi:10.3389/fonc.2019.01011)
Supplement: Supplementary file 1 [file Data_Sheet_1.zip › SupplementaryMaterials/Figure_S1.pdf]

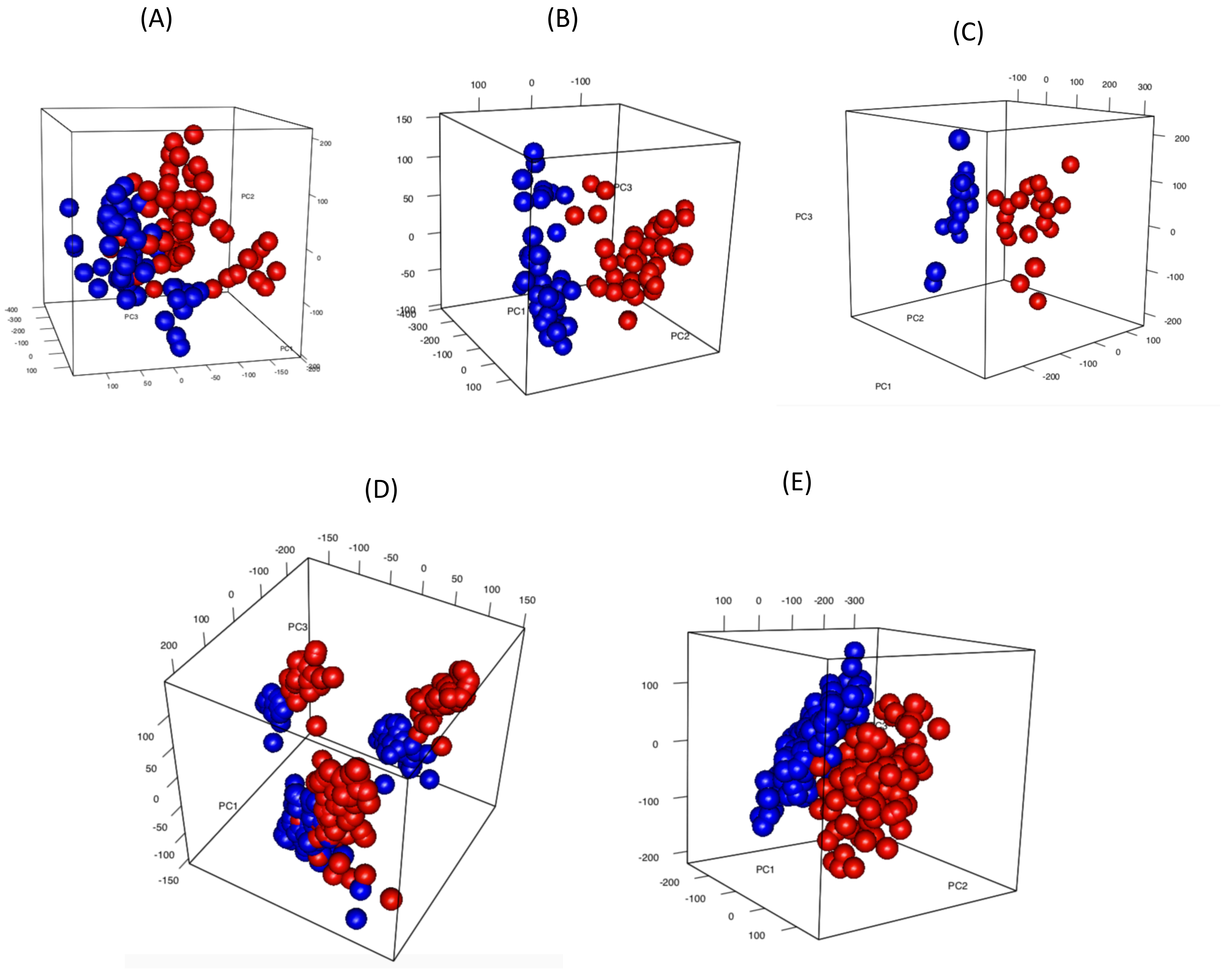

Figure S1: Principal component analysis of microarray expression data of NSCLC cancer and normal. The figure showed first three PCA data (PC1, PC2 and PC3) which reveals that the gene expression patterns of NSCLC and normal are relatively distinct in each GSEs: GSE19804 (A); GSE18842 (B); GSE27262 (C). PCA before (D); and after removing batch effect (E) with ComBat of sva in R. NSCLC cancer is in red; while normal is in blue.
