# Supplementary material for: Integrated Network Analysis Reveals FOXM1 and MYBL2 as Key Regulators of Cell Proliferation in Non-small Cell Lung Cancer
Source: Front Oncol. 2019 Oct 15;9:1011. doi: 10.3389/fonc.2019.01011 (PMC6804573; doi:10.3389/fonc.2019.01011)
Supplement: Supplementary file 1 [file Data_Sheet_1.zip › SupplementaryMaterials/Figure_S5.pdf]

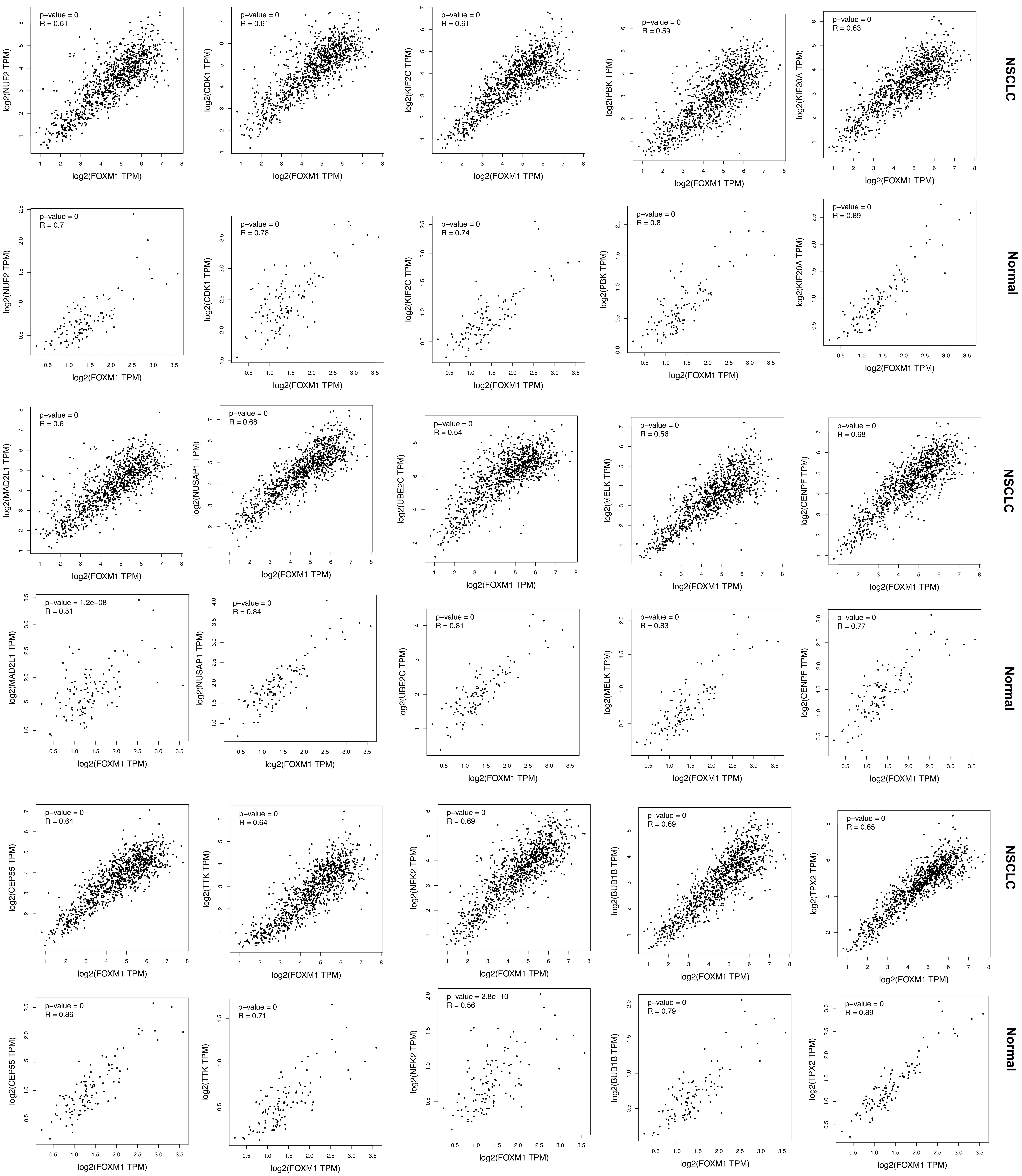


**Figure S5:** Correlation analysis of expression of genes in *Cluster 1* and its TFs. Expression of gene is on Y-axis while TF is on X-axis.

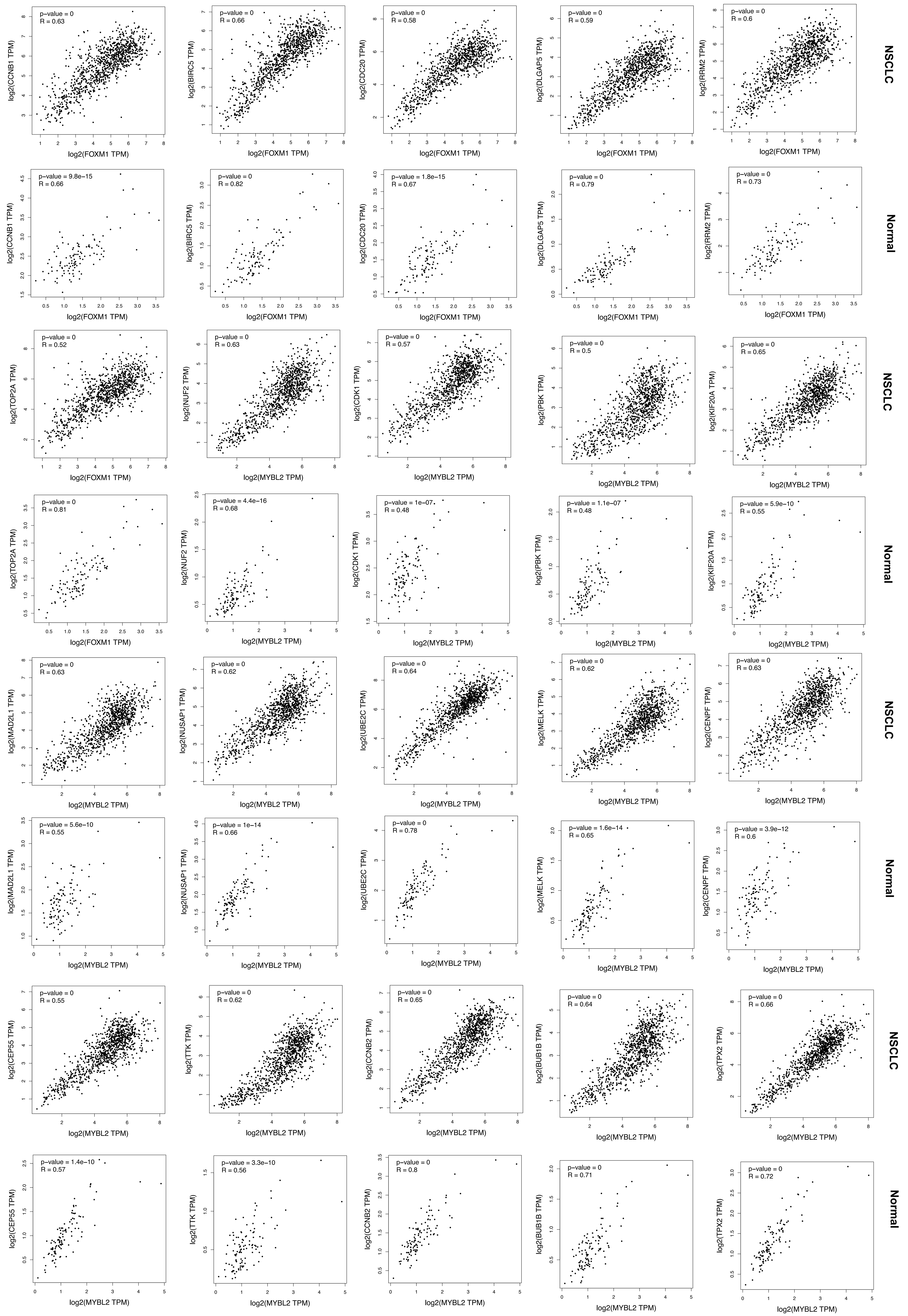

p−value = 0
R = 0.63
log2(CCNB1 TPM)
log2(FOXM1 TPM)
p−value = 0
R = 0.66
log2(BIRC5 TPM)
log2(FOXM1 TPM)
p−value = 0
R = 0.58
log2(CDC20 TPM)
log2(FOXM1 TPM)
p−value = 0
R = 0.59
log2(DLGAP5 TPM)
log2(FOXM1 TPM)
p−value = 0
R = 0.6
log2(RRM2 TPM)
log2(FOXM1 TPM)
NSCLC
p−value = 9.8e−15
R = 0.66
log2(CCNB1 TPM)
log2(FOXM1 TPM)
p−value = 0
R = 0.82
log2(BIRC5 TPM)
log2(FOXM1 TPM)
p−value = 1.8e−15
R = 0.67
log2(CDC20 TPM)
log2(FOXM1 TPM)
p−value = 0
R = 0.79
log2(DLGAP5 TPM)
log2(FOXM1 TPM)
p−value = 0
R = 0.73
log2(RRM2 TPM)
log2(FOXM1 TPM)
Normal
p−value = 0
R = 0.52
log2(TOP2A TPM)
log2(FOXM1 TPM)
p−value = 0
R = 0.63
log2(NUF2 TPM)
log2(MYBL2 TPM)
p−value = 0
R = 0.57
log2(CDK1 TPM)
log2(MYBL2 TPM)
p−value = 0
R = 0.5
log2(PBK TPM)
log2(MYBL2 TPM)
p−value = 0
R = 0.65
log2(KIF20A TPM)
log2(MYBL2 TPM)
NSCLC
p−value = 0
R = 0.81
log2(TOP2A TPM)
log2(FOXM1 TPM)
p−value = 4.4e−16
R = 0.68
log2(NUF2 TPM)
log2(MYBL2 TPM)
p−value = 1e−07
R = 0.48
log2(CDK1 TPM)
log2(MYBL2 TPM)
p−value = 1.1e−07
R = 0.48
log2(PBK TPM)
log2(MYBL2 TPM)
p−value = 5.9e−10
R = 0.55
log2(KIF20A TPM)
log2(MYBL2 TPM)
Normal
p−value = 0
R = 0.63
log2(MAD2L1 TPM)
log2(MYBL2 TPM)
p−value = 0
R = 0.62
log2(NUSAP1 TPM)
log2(MYBL2 TPM)
p−value = 0
R = 0.64
log2(UBE2C TPM)
log2(MYBL2 TPM)
p−value = 0
R = 0.62
log2(MELK TPM)
log2(MYBL2 TPM)
p−value = 0
R = 0.63
log2(CENPF TPM)
log2(MYBL2 TPM)
NSCLC
p−value = 5.6e−10
R = 0.55
log2(MAD2L1 TPM)
log2(MYBL2 TPM)
p−value = 1e−14
R = 0.66
log2(NUSAP1 TPM)
log2(MYBL2 TPM)
p−value = 0
R = 0.78
log2(UBE2C TPM)
log2(MYBL2 TPM)
p−value = 1.6e−14
R = 0.65
log2(MELK TPM)
log2(MYBL2 TPM)
p−value = 3.9e−12
R = 0.6
log2(CENPF TPM)
log2(MYBL2 TPM)
Normal
p−value = 0
R = 0.55
log2(CEP55 TPM)
log2(MYBL2 TPM)
p−value = 0
R = 0.62
log2(TTK TPM)
log2(MYBL2 TPM)
p−value = 0
R = 0.65
log2(CCNB2 TPM)
log2(MYBL2 TPM)
p−value = 0
R = 0.64
log2(BUB1B TPM)
log2(MYBL2 TPM)
p−value = 0
R = 0.66
log2(TPX2 TPM)
log2(MYBL2 TPM)
NSCLC
p−value = 1.4e−10
R = 0.57
log2(CEP55 TPM)
log2(MYBL2 TPM)
p−value = 3.3e−10
R = 0.56
log2(TTK TPM)
log2(MYBL2 TPM)
p−value = 0
R = 0.8
log2(CCNB2 TPM)
log2(MYBL2 TPM)
p−value = 0
R = 0.71
log2(BUB1B TPM)
log2(MYBL2 TPM)
p−value = 0
R = 0.72
log2(TPX2 TPM)
log2(MYBL2 TPM)
Normal

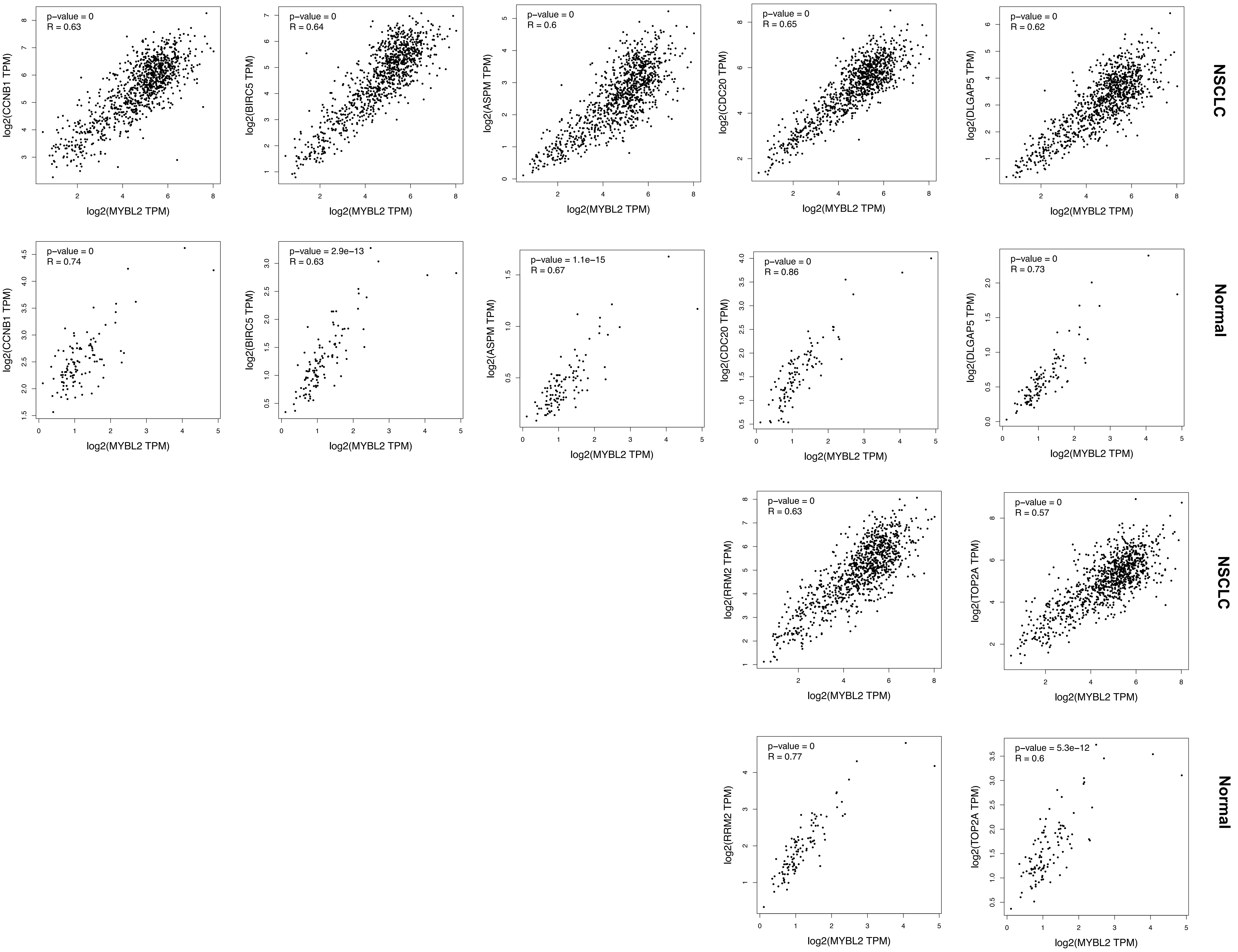

NSCLC
Normal
NSCLC
Normal
p−value = 0
R = 0.63
log2(CCNB1 TPM)
log2(MYBL2 TPM)
p−value = 0
R = 0.64
log2(BIRC5 TPM)
p−value = 0
R = 0.6
log2(ASPM TPM)
p−value = 0
R = 0.65
log2(CDC20 TPM)
p−value = 0
R = 0.62
log2(DLGAP5 TPM)
p−value = 0
R = 0.74
p−value = 2.9e−13
R = 0.63
p−value = 1.1e−15
R = 0.67
p−value = 0
R = 0.86
p−value = 0
R = 0.73
p−value = 0
R = 0.63
log2(RRM2 TPM)
p−value = 0
R = 0.57
log2(TOP2A TPM)
p−value = 0
R = 0.77
p−value = 5.3e−12
R = 0.6
