# Supplementary material for: Integrated Network Analysis Reveals FOXM1 and MYBL2 as Key Regulators of Cell Proliferation in Non-small Cell Lung Cancer
Source: Front Oncol. 2019 Oct 15;9:1011. doi: 10.3389/fonc.2019.01011 (PMC6804573; doi:10.3389/fonc.2019.01011)
Supplement: Supplementary file 1 [file Data_Sheet_1.zip › SupplementaryMaterials/Figure_S4.pdf]

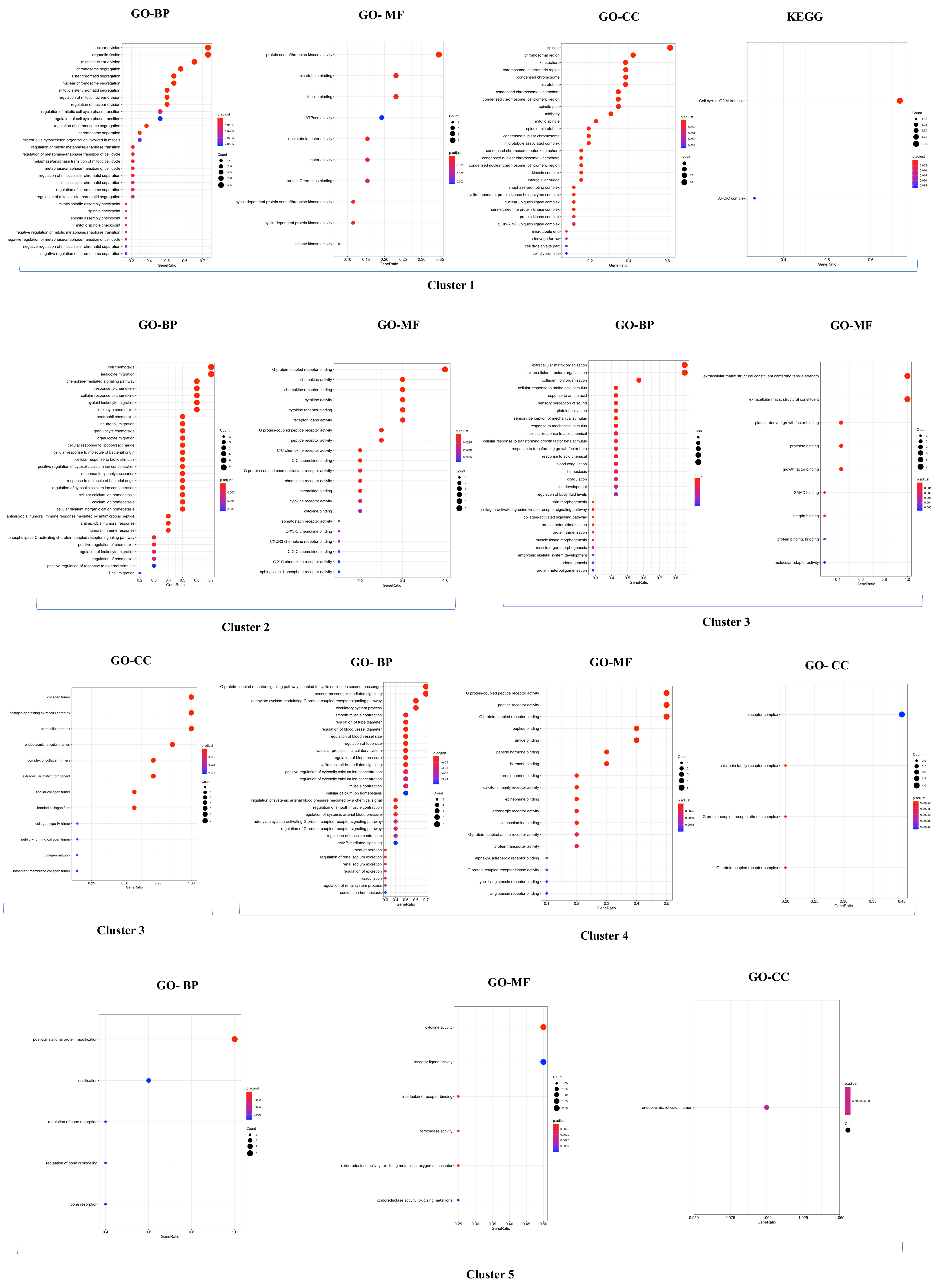

Figure S4: Functional annotation of MCODE five top clusters extracted from NSCLC Network. Cluster 1, Cluster 2, Cluster 3, Cluster 4, and Cluster 5. GO: Gene Ontology; BP: Biological Processes; MF: Molecular Function; CC: Cell Component; KEGG: Kyoto Encyclopedia of Genes and Genomes. Dot plot is not available for Cluster having no significant functional enrichment.
